# Early Growth Response 1 Strengthens Pol-III-Directed Transcription and Transformed Cell Proliferation by Controlling PTEN/AKT Signalling Activity

**DOI:** 10.3390/ijms23094930

**Published:** 2022-04-29

**Authors:** Zhongyu Wu, Liyun Huang, Shasha Zhao, Juan Wang, Cheng Zhang, Xiaoye Song, Qiyue Chen, Jiannan Du, Deen Yu, Xiaomeng Sun, Yue Zhang, Wensheng Deng, Shihua Zhang, Huan Deng

**Affiliations:** 1 School of Life Science and Health, Wuhan University of Science and Technology, Wuhan 430072, China; wuzhongyu155@163.com (Z.W.); hunagly0802@163.com (L.H.); zhaoshasha@wust.edu.cn (S.Z.); wangjuan@wust.edu.cn (J.W.); zhangcheng10299@163.com (C.Z.); sxyyezi@126.com (X.S.); chenqiyue202101@163.com (Q.C.); dujiannan41@163.com (J.D.); yde0315@163.com (D.Y.); sunxiaomeng0423@163.com (X.S.); yuezhang1388@163.com (Y.Z.); zhangshihua@wust.edu.cn (S.Z.); 2 School of Materials and Metallurgy, Wuhan University of Science and Technology, Wuhan 430072, China

**Keywords:** EGR1, RNA polymerase III, transcription, PTEN/AKT signalling, cell proliferation

## Abstract

RNA polymerase III (Pol III) products play essential roles in ribosome assembly, protein synthesis, and cell survival. Deregulation of Pol-III-directed transcription is closely associated with tumorigenesis. However, the regulatory pathways or factors controlling Pol-III-directed transcription remain to be investigated. In this study, we identified a novel role of EGR1 in Pol-III-directed transcription. We found that Filamin A (FLNA) silencing stimulated EGR1 expression at both RNA and protein levels. EGR1 expression positively correlated with Pol III product levels and cell proliferation activity. Mechanistically, EGR1 downregulation dampened the occupancies of Pol III transcription machinery factors at the loci of Pol III target genes. Alteration of EGR1 expression did not affect the expression of p53, c-MYC, and Pol III general transcription factors. Instead, EGR1 activated RhoA expression and inhibited PTEN expression in several transformed cell lines. We found that PTEN silencing, rather than RhoA overexpression, could reverse the inhibition of Pol-III-dependent transcription and cell proliferation caused by EGR1 downregulation. EGR1 could positively regulate AKT phosphorylation levels and is required for the inhibition of Pol-III-directed transcription mediated by FLNA. The findings from this study indicate that EGR1 can promote Pol-III-directed transcription and cell proliferation by controlling the PTEN/AKT signalling pathway.

## 1. Introduction

Early growth response 1 (EGR1) is a zinc-finger transcription factor that can bind a DNA consensus sequence GCG (G/T)GGGGCG at its target genes. EGR1 is involved in the regulation of several important cellular processes, including cell proliferation, cell invasion and migration, apoptosis, and angiogenesis [1,2,3]. Despite a low level of expression in many cell types, EGR1 expression can be induced by extracellular growth factors, cytokines, radiation, and mechanical stress [2,4,5,6,7]. It has been shown that EGR1 expression is activated by the MAPK signalling pathway comprising three basic family members: p38MAPK, ERK1/2, and JNK [1,2]. Furthermore, EGR1 gene expression is also controlled by the regulatory elements of its promoter, including serum response elements (SREs), cAMP regulatory elements (CREs), AP1 binding sites, and Sp1 binding sites [1,2,8,9,10]. EGR1 directly regulates the expression of several tumour suppressors such as p53, PTEN, TGF-β 1, and fibronectin by binding to their promoters, which can induce cell cycle arrest and apoptosis [3,11,12,13,14,15]. In pancreatic cancers, EGR1 binds to the Bax gene promoter to activate BAX expression, which subsequently leads to cancer cell apoptosis [16]. Apart from acting as a tumour suppressor, EGR1 has also been shown to promote cancer development, including prostate, lung, and gastric cancers [17,18,19]. Recently, EGR1 has been found to interact with a LncRNA SLNCR and an androgen receptor to promote oncogenesis for melanomas [20]. However, the molecular basis of EGR1-dependent promotion of oncogenesis is not fully understood.

Human RNA polymerase III (Pol III) is responsible for the synthesis of 5S rRNA, tRNAs, U6 RNA, 7SL RNA, and other noncoding RNAs. These Pol III products are essential to maintaining the fundamental activities in cells, including protein synthesis, protein transportation, RNA splicing, and metabolism, and these activities also control cell survival and growth [21,22,23,24,25]. Deregulation of Pol-III-directed transcription and abnormal expression of Pol-III-related factors are associated with tumorigenesis [26]. It has been shown that TFIIIB and Pol III products have an aberrantly high expression in many cancers. For example, high levels of tRNA expression in breast cancer and myelomas tissues have been observed when compared with normal tissues [27,28]. Pol III transcription initiation requires general transcription factors and Pol III to assemble at the loci of its target genes, where general factors play pivotal roles in transcription initiation. In addition to general transcription factors, Pol-III-mediated transcription is tightly controlled by oncogenic factors, tumour suppressors, and chromatin remodelling factors [26,29], and several signalling pathways, including TORC1, RAS/ERK, JNK, and PTEN/AKT, have been confirmed to regulate Pol-III-directed transcription and other biological activities [30,31,32,33,34,35]. However, whether other signalling factors are required for the regulation of Pol-III-directed transcription remains to be identified. 

Cytoskeletal Filamin A (FLNA) is an actin-binding protein that maintains a three-dimensional cytoskeleton network in human cells, regulating numerous cellular processes such as cell proliferation, cell invasion, and migration, signal transduction, transcription, etc. [36,37]. It has been reported that FLNA silencing stimulated Pol-III-directed transcription in transformed cell lines [38]. However, the mechanism underlying this event is not fully understood. In this study, we found that EGR1 can act as a positive factor to regulate Pol III product expression and cell proliferation, and EGR1 is also required for the inhibition of Pol-III-directed transcription mediated by FLNA. We explored the molecular mechanism by which EGR1 modulates Pol-III-directed transcription.

## 2. Results

### 2.1. FLNA Downregulation Stimulates EGR1 Expression in Transformed Cell Lines

To uncover the mechanism by which FLNA modulates Pol-III-directed transcription, recently, we performed RNA-seq analysis using SaOS2 FLNA-depleted cell line and its control cell line, and the clean data have been submitted to the NCBI repository online (Submission ID: SUB9541028, https://www.ncbi.nlm.nih.gov/Traces/study/?acc=PRJNA726417) (accessed on 4 May 2021). Analysis of differential expression genes (note that the differential expression genes (DEGs) are the genes when the expression difference between controls and treatments is over twofold) revealed that FLNA depletion significantly activated EGR1 mRNA expression in SaOS2 cells (Figure S1A,B). To confirm this observation, we examined EGR1 expression in SaOS2 FLNA-depleted cells using RT-qPCR and Western blotting. As expected, FLNA knockdown enhanced EGR1 expression at both RNA and protein levels (Figure S1C–E). To determine whether this result can be reproduced in other cell types, we generated 293T and HeLa cell lines stably expressing FLNA shRNA or control shRNA, and EGR1 expression was examined by RT-qPCR and Western blotting. Data showed that FLNA silencing indeed stimulated EGR1 expression in these cell types (Figure S1F–K). These results suggest that FLNA can inhibit EGR1 expression at both RNA and protein levels in several transformed cell lines.

### 2.2. EGR1 Acts as an Activator to Regulate Transcription of Most Pol III Target Genes Tested in This Study

**Figure 1:**
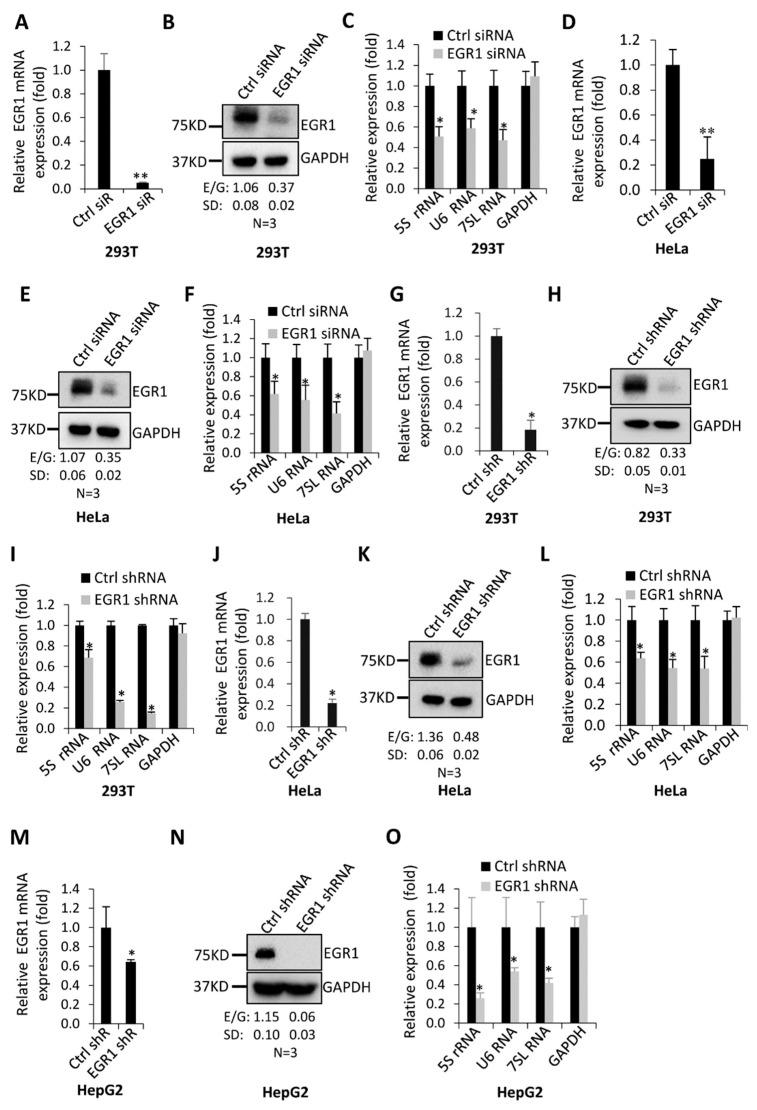
EGR1 downregulation inhibited the expression of Pol III products: (**A**–**C**) EGR1 siRNA transfection reduced expression of Pol III products in 293T cells. In this study, 293T cells were seeded in 12-well plates and transfected with EGR1 siRNA. After 48 h, EGR1 expression was monitored by RT-qPCR (**A**) and Western blotting (**B**). Pol III products were analysed by RT-qPCR (**C**); (**D**–**F**) EGR1 siRNA transfection inhibited the expression of Pol III products in HeLa cells. HeLa cells were transfected as for 293T cells. EGR1 expression was examined by RT-qPCR (**D**) and Western blotting (**E**), and Pol III products were analysed by RT-qPCR (**F**); (**G**–**I**) EGR1 shRNA stable expression impeded Pol-III-directed transcription in 293T cells. Briefly, 293T cell lines stably expressing EGR1 shRNA or control shRNA were generated using a lentiviral infection system. EGR1 expression was determined by RT-qPCR (**G**) and Western blotting (**H**), and Pol III products were detected by RT-qPCR (**I**); (**J**–**L**) EGR1 shRNA stable expression repressed Pol-III-directed transcription in HeLa cells. HeLa cell lines stably expressing EGR1 shRNA or control shRNA were generated using a lentiviral infection system. EGR1 expression was determined by RT-qPCR (**J**) and Western blot (**K**), and Pol III products were analysed by RT-qPCR (**L**); (**M**–**O**) EGR1 shRNA stable expression inhibited the expression of Pol III products in HepG2 cells. HepG2 cell lines stably expressing EGR1 shRNA or control shRNA were generated with a similar procedure as that for 293T cells. EGR1 expression was examined by RT-qPCR (**M**) and Western blotting (**N**), and Pol III products were monitored by RT-qPCR (**O**). E/G in (**B**,**E**,**H**,**K**,**N**) represents the ratio of the EGR1 (E) intensity to the GAPDH (G) intensity (*n* = 3); SD, standard deviation. Each column in the bar graphs represents the mean ± SD of three biological replicates (*n* = 3). *, *p* < 0.05; **, *p* < 0.01. The *p* values of different genes were obtained by Student’s *t*-test, performed with control and treatment groups.

As demonstrated above, EGR1 expression was stimulated by FLNA downregulation, whereas FLNA silencing has previously been shown to activate Pol-III-directed transcription [38]. Whether alteration of EGR1 expression is associated with the regulation of Pol-III-directed transcription has not been investigated. To address this question, we first tested the effect of EGR1 downregulation on the expression of Pol III products, including 5S rRNA, U6 RNA, and 7SL RNA expression. In total, 293T cells were initially transfected with EGR1 siRNA fragment, and EGR1 expression was monitored by RT-qPCR and Western blotting. As illustrated in Figure 1A,B, EGR1 expression was downregulated by EGR1 siRNA transfection. Strikingly, the EGR1 knockdown severely dampened the expression of Pol III products (Figure 1C). A similar experiment was performed using HeLa cells transiently transfected with EGR1 siRNA, and the result was in agreement with that obtained from 293T cells (Figure 1D–F). To confirm these results, we next generated 293T, HeLa, and HepG2 cell lines stably expressing EGR1 shRNA or control shRNA, using a lentiviral infection system. Both RT-qPCR and Western blot data showed that EGR1 expression was inhibited by the EGR1 shRNA stable expression (Figure 1G,H,J,K,M,N). Analysis of Pol III products revealed that EGR1 shRNA stable expression impeded the expression of all Pol III products tested in these assays, although a slight discrepancy among Pol III products or cell types was observed (Figure 1I,L,O). Collectively, these data indicate that EGR1 is required for Pol-III-directed transcription and may positively regulate this process. 

**Figure 2:**
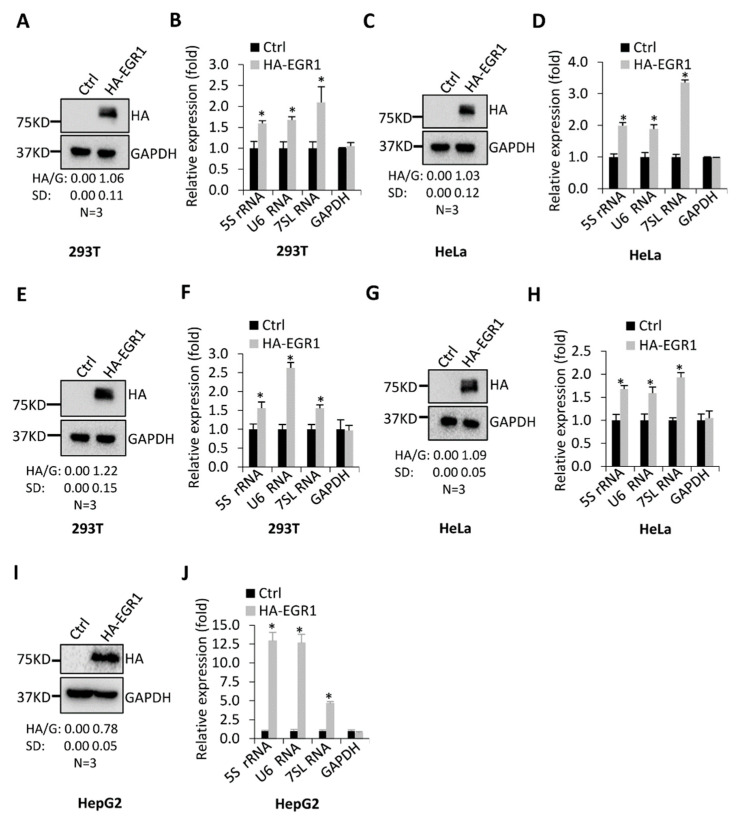
EGR1 overexpression activated Pol-III-directed transcription: (**A**) Western blotting was used to detect HA–EGR1 expression in the 293T cells transiently transfected with the HA–EGR1-expressing vectors; (**B**) Pol III products were analysed by RT-qPCR using the 293T cells obtained in (**A**); (**C**) HA–EGR1 expression was analysed by Western blotting using the HeLa cells transiently transfected with the HA–EGR1-expressing vectors; (**D**) Pol III products were detected by RT-qPCR using the HeLa cells obtained in (**C**); (**E**) Western blotting was used to determine the generation of the 293T cell line stably expressing HA–EGR1 and its control cell line; (**F**) RT-qPCR was used to analyse the expression of Pol III products in the 293T cell line stably expressing HA–EGR1 and its control cell line; (**G**) Western blotting was used to determine a HeLa cell line stably expressing HA–EGR1 and its control cell line; (**H**) RT-qPCR data showing expression of Pol III products in the HeLa cell line stably expressing HA–EGR1 and its control cell line; (**I**) Western blot data showing expression of EGR1 in the HeLa cell line stably expressing HA–EGR1 and its control cell line; (**J**) expression of Pol III products in the HeLa cell line stably expressing HA–EGR1 and its control cell line was monitored by RT-qPCR. In (**A**,**C**,**E**,**G**,**I**), H/G represents the ratio of the HA–EGR1 (H) intensity to the GAPDH (G) intensity (*n* = 3). Each column in the bar graphs represents the mean ± SD of three biological replicates (*n* = 3). *, *p* < 0.05. The *p* values of different genes were obtained by Student’s *t*-test, performed with control and treatment groups.

To confirm the positive role of EGR1 in the expression of Pol III products, we performed transient transfect assays using 293T or HeLa cells and HA–EGR1-expressing vectors. After selection by puromycin, cells were harvested and lysed for the analysis of HA–EGR1 expression by Western blot. Western blot showed that HA–EGR1 fusion protein was expressed in these cell types after transfection of the HA–EGR1-expressing vectors (Figure 2A,C). Notably, HA–EGR1 expression in both 293T and HeLa cells enhanced the synthesis of 5S rRNA, U6 RNA, and 7SL RNA (Figure 2B,D). To validate this observation, we generated 293T, HeLa, and HepG2 cell lines stably expressing HA–EGR1 and their control cell lines using a lentiviral infection system. Western blot revealed that the cell lines stably expressing HA–EGR1 fusion protein had been established (Figure 2E,G,I). Expectedly, EGR1 overexpression enhanced the expression of Pol III products, confirming that EGR1 can activate Pol-III-directed transcription.

**Figure 3:**
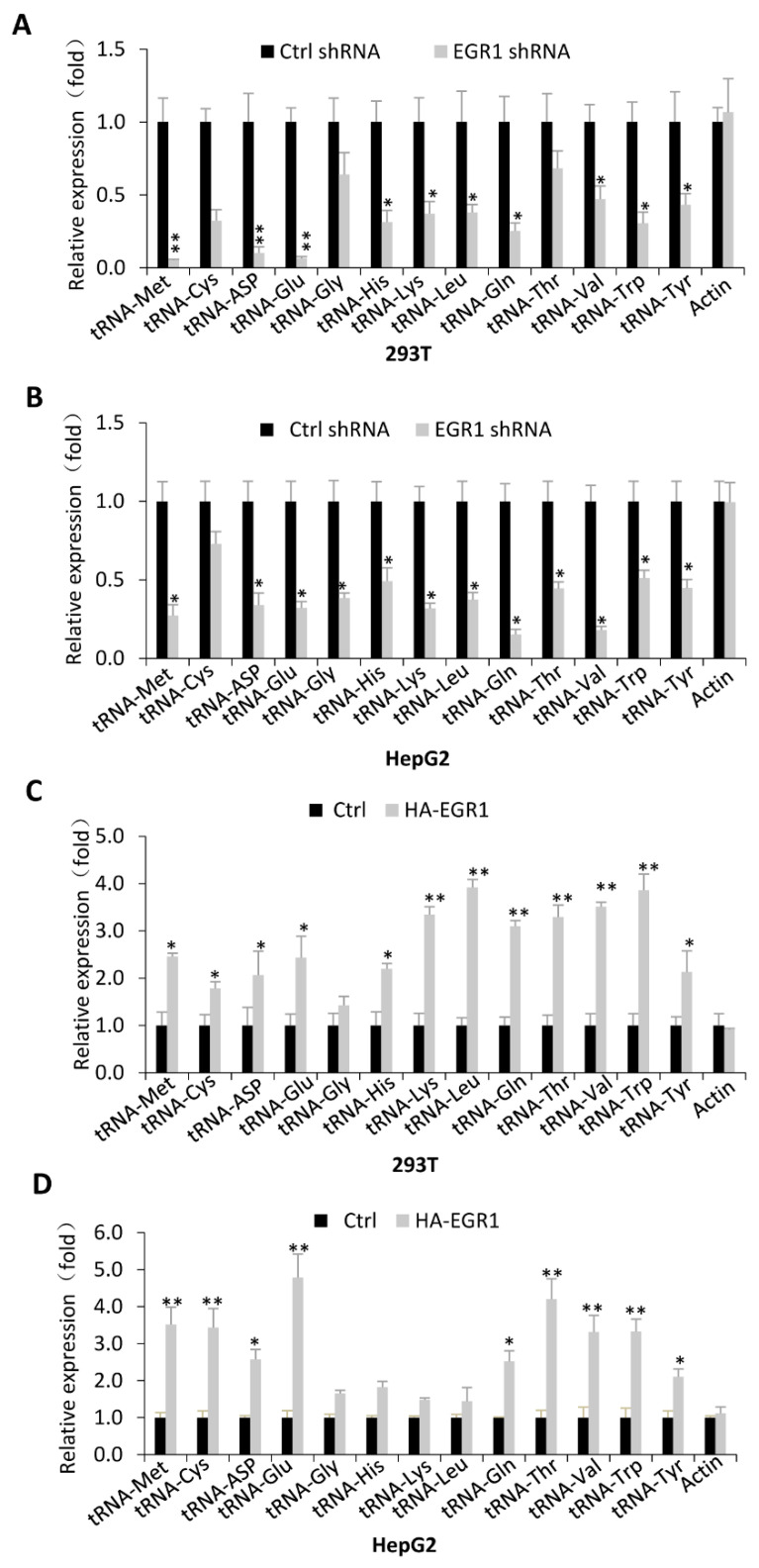
EGR1 positively regulated the expression of most tRNA genes tested in the assays: (**A**) transfer RNA (tRNA) gene expression was analysed by RT-qPCR using 293T cell lines stably expressing EGR1 shRNA and control shRNA; (**B**) expression of tRNA genes in HepG2 cell lines stably expressing EGR1 shRNA or control shRNA was monitored by RT-qPCR. (**C**) RT-qPCR was used to analyse the expression of tRNA genes in a 293T cell line stably expressing HA–GER1 and its control cell line; (**D**) expression of tRNA genes in the HepG2 cell line stably expressing HA–GER1 and its control cell line was detected by RT-qPCR. Each column in graphs represents the mean ± SD of three biological replicates (*n* = 3). *, *p* < 0.05; **, *p* < 0.01. The *p* values of different genes were obtained by Student’s *t*-test, performed with control and treatment groups.

Transfer RNA molecules are a class of Pol III products that are involved in protein synthesis, cell growth, and metabolism. How EGR1 regulates tRNA expression was unclear. Using the stable cell lines established above, we randomly selected and analysed the expression of a subset of tRNA genes by RT-qPCR. Interestingly, EGR1 silencing in 293T and HepG2 cells repressed the expression of most tRNA genes examined (Figure 3A,B). In contrast, EGR1 overexpression in these cell types enhanced the expression of most tRNA genes tested in the assays (Figure 3C,D). However, we observed that some of the tRNA genes showed less sensitivity to EGR1 expression change and displayed differential expression between two cell types (Figure 3), suggesting that EGR1 regulates the synthesis of tRNA products in a cell type-specific manner. It is possible that different cell types require different quantities for each type of tRNA because protein components among cell types are distinct. Taken together, EGR1 can act as an activator to regulate the transcription of most Pol III target genes tested in this study.

### 2.3. The Activation of Pol-III-Directed Transcription Mediated by EGR1 Contributes to the Promotion of Cell Proliferation

**Figure 4:**
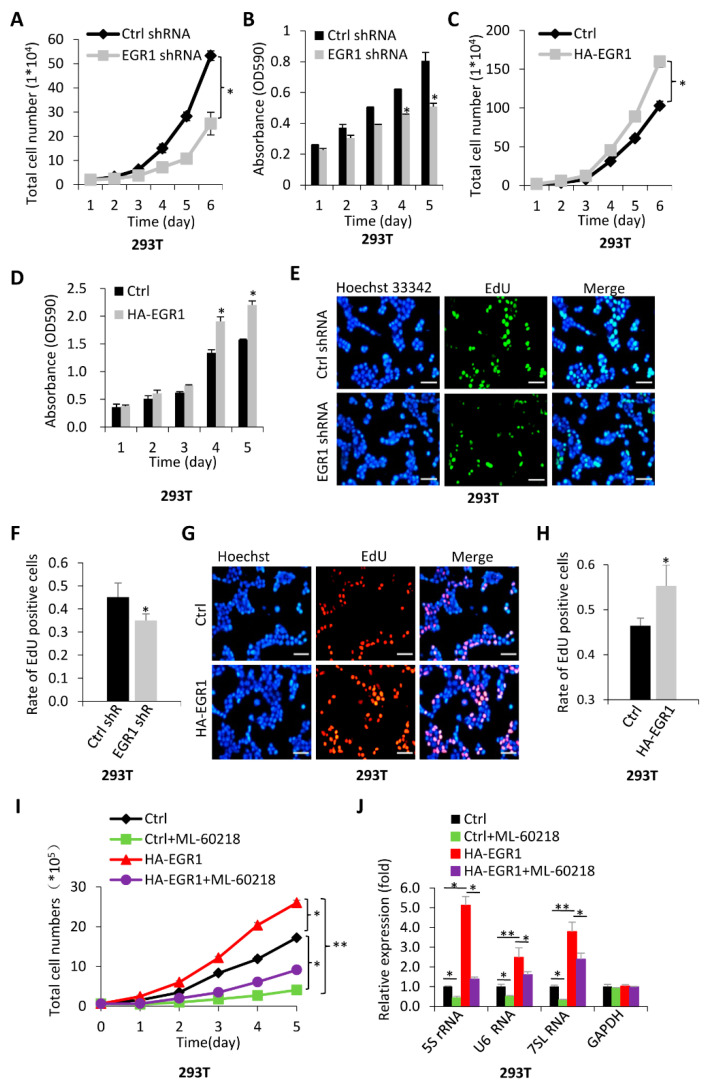
EGR1 may promote cell proliferation by activating Pol-III-directed transcription: (**A**,**B**) EGR1 knockdown impeded 293T cell proliferation. Briefly, 293T cell lines stably expressing EGR1 shRNA and control shRNA were cultured and subjected to proliferation assays using cell counting (**A**) and MTT assays (**B**); (**C**,**D**) EGR1 overexpression enhanced 293T cell proliferation activity. In this study, 293T cell lines stably expressing HA–EGR1 and its control cell line were cultured and subjected to proliferation assays using cell counting (**C**) and MTT assays (**D**); (**E**,**F**) EdU assay results showing the effect of EGR1 knockdown on 293T cell proliferation. The representative images obtained from EdU assays are presented in (**E**). (**F**) represents a statistical result from minimal 5 images; (**G**,**H**) EdU assay results showing the effect of HA–EGR1 expression on 293T cell proliferation. The representative images obtained from EdU assays are presented in (**G**). (**H**) represents a statistical result from minimal 5 images; (**I**) the presence of ML-60218 repressed the enhancement of cell proliferation caused by EGR1 overexpression. Cell counting was performed using 293T cell lines as indicated; (**J**) the presence of ML-60218 inhibited the activation of Pol III transcription caused by EGR1 overexpression. Pol III products were analysed by RT-qPCR using the total RNA extracted from 293T cell lines as indicated. The scale bars in EdU images represent 50 μm. Each point or column in graphs represents the mean ± SD of three biological replicates (*n* = 3). *, *p* < 0.05; **, *p* < 0.01. *p* values for (**A**–**D**) were obtained by two-way ANOVA; *p* values for (**F**,**H**,**I**) were obtained using Student’s *t*-test, performed with control and treatment samples; *p* values for (**J**) were obtained by one-way ANOVA, followed by Bonferroni test, using a comparison of two groups among multiple groups.

Pol III products are essential to several cellular processes, including protein synthesis, protein transportation, RNA splicing, cell growth, and metabolism [21,23]. Pol III product levels closely correlate with cell growth. Thus, we first examined the effect of EGR1 expression change on 293T cell proliferation activity. Intriguingly, EGR1 silencing dampened the proliferation activity of 293T cells. In contrast, EGR1 overexpression enhanced 293T cell proliferation (Figure 4A–D). Using the same methods as those utilised for 293T cells, we examined the proliferation activity of HeLa and HepG2 cell lines stably expressing EGR1 shRNA or HA–EGR1 and their control cell lines, and the results were consistent with those obtained with 293T cells (Figure S2). Furthermore, 5-ethynyl-2’-deoxyuridine (EdU) can be easily incorporated into the genomic DNA during cell cycles and is widely used in cell proliferation assays. Thus, we analysed 293T cell proliferation activity using EdU assays. We found that EGR1 silencing decreased the rate of EdU positive cells (Figure 4E,F), whereas EGR1 overexpression increased the rate of EdU-labelled cells (Figure 4G,H). Furthermore, comparable results to those from 293T cells were obtained when HeLa and HepG2 cell lines were used for EdU assays (Figure S3). These results confirm that EGR1 promotes the proliferation activity of 293T, HeLa, and HepG2 cells.

We demonstrated that EGR1 expression positively correlates with Pol-III-directed transcription and cell proliferation activity. We next determined whether the enhancement of cell proliferation is associated with the activation of Pol III products mediated by EGR1. To this end, we performed cell proliferation assays in the presence or absence of Pol-III-specific inhibitor ML-60218 using a 293T cell line stably expressing HA–EGR1 and its control cell line. Figure 4I shows that the presence of ML-60218 inhibited 293T cell proliferation, whereas EGR1 overexpression enhanced 293T cell proliferation. Interestingly, the presence of ML-60218 inhibited the enhancement of proliferation activity caused by EGR1 overexpression (Figure 4I). RT-qPCR showed that the activation of Pol-III-directed transcription caused by EGR1 overexpression was also repressed in the presence of ML-60218 (Figure 4J). Similar assays were performed using HeLa and HepG2 cell lines stably expressing HA–EGR1 and their control cell lines, and the results were consistent with those obtained in 293T cells (Figure S4). These results indicate that the activation of Pol III transcription mediated by EGR1 contributes to the enhancement of cell proliferation.

### 2.4. EGR1 Regulates PTEN and RhoA Expression but Does Not Affect the Expression of Pol III Transcription Factors

**Figure 5:**
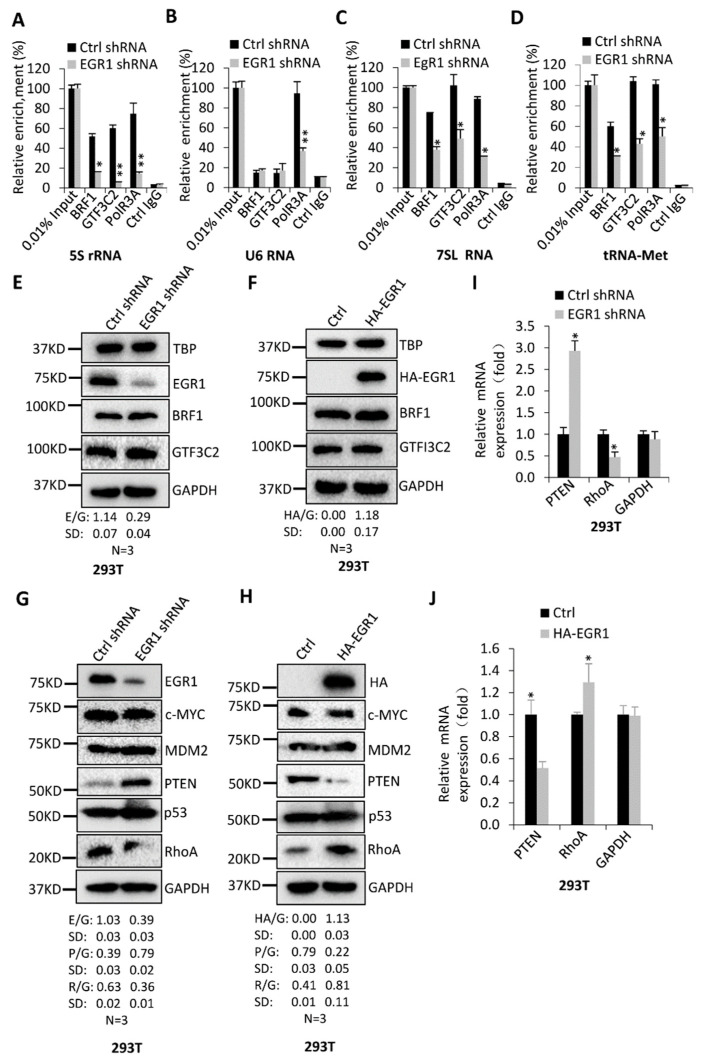
Alteration of EGR1 expression affected PTEN and RhoA expression: (**A**–**D**) ChIP assay results showing the effect of EGR1 on the occupancies of Pol III transcription machinery factors at the promoters of Pol III target genes. ChIP assays were performed using 293T cell lines stably expressing EGR1 shRNA and control shRNA and the antibodies against the factors, as indicated. The relative quantities of the DNA fragments at the loci of 5S rRNA (**A**), U6 RNA (**B**), 7SL RNA (**C**), and tRNA–Met (**D**) genes were detected by RT-qPCR. Relative enrichment was obtained by comparing the relative DNA quantity in a 1 μL ChIP DNA sample to that of 0.01% input; (**E**,**F**) EGR1 expression change did not affect the expression of TBP, BRF1, and GTF3C2 in 293T cells. Western blotting was performed using 293T cell lines stably expressing EGR1 shRNA (**E**) and HA–EGR1 (**F**) and their control cell lines; (**G**,**H**) expression of oncogenic proteins, suppressors. and signalling factors in the 293T cell lines with EGR1 depletion (**G**) or overexpression (**H**) was examined by Western blotting; (**I**,**J**) expression of PTEN and RhoA mNA was monitored by RT-qPCR using the 293T cell line with EGR1 depletion or overexpression. E/G, H/G, P/G, and R/G in Western blot quantification, respectively represent the ratio of EGR1 (E), HA–EGR1 (H), PTEN (P), and RhoA (R) intensities to the GAPDH (G) intensity (*n* = 3). Each column in the bar graphs represents the mean ± SD of three biological replicates (*n* = 3). *, *p* < 0.05; **, *p* < 0.01. The *p* values of different genes or factors were obtained by Student’s *t*-test, performed with control and treatment groups.

Pol-III-mediated transcription is directly controlled by the assembly of the Pol III transcription machinery at its target genes. To uncover the mechanism by which EGR1 regulates Pol-III-directed transcription, we first performed ChIP assays using 293T cell lines stably expressing EGR1 shRNA or control shRNA and the antibodies against the factors of Pol III transcription machinery, as indicated. ChIP-qPCR data revealed that EGR1 downregulation reduced the occupancies of BRF1, GTF3C2, and POLR3A at the promoters of 5S rRNA, 7SL RNA, and tRNA-Met genes. However, the occupancies of BRF1 and GTF3C2 at the promoter of U6 RNA showed a level that is similar to control IgG and were not significantly affected by EGR1 depletion when compared with the occupancy of POLR3A at this promoter (Figure 5A–D). This result is reasonable because BRF1 and GTF3C2 are not essential to U6 RNA expression [21,22]. Next, we determined how EGR1 silencing affected Pol III transcription machinery assembly at the promoters of Pol III target genes. General transcription factors can directly modulate Pol III transcription assembly at the promoters of the Pol III target genes; thus, we first analysed the expression of Pol III transcription factor subunits by Western blotting using 293T cell lines with EGR1 depletion or overexpression. Unexpectedly, both EGR1 downregulation and upregulation did not affect TBP, BRF1, and GTF3C2 expression, (Figure 5E,F). Consistent results were obtained when these factors were examined in HeLa and HepG2 cells (Figure S5A–D). These data suggest that EGR1 cannot modulate the recruitment of Pol III transcription machinery at its target loci by directly controlling the expression of general transcription factors. Oncogenic factors, tumour suppressors, and signalling factors have been shown to indirectly regulate Pol-III–directed transcription [26]. Thus, the expression of these factors was determined by Western blotting using the cell lines with EGR1 silencing or overexpression. Interestingly, EGR1 downregulation in 293T and HepG2 cells activated PTEN expression but reduced RhoA expression (Figure 5G and Figure S5E). In contrast, EGR1 overexpression in these cell types inhibited PTEN expression but enhanced RhoA expression (Figure 5H and Figure S5F). Expression of other factors, including p53, MDM2, and c-MYC, was not affected by EGR1 expression change. To understand how EGR1 regulates RhoA and PTEN expression, we analysed the mRNA levels of these two factors by RT-qPCR using 293T cell lines with EGR1 silencing or overexpression. Interestingly, alteration of EGR expression affected RNA expression levels of these two factors, and the results are consistent with those of protein expression (Figure 5I,J), indicating that EGR1 regulates the expression of PTEN and RhoA at both RNA and protein levels. These data suggest that EGR1 may regulate Pol-III-directed transcription by affecting the expression of PTEN and RhoA.

### 2.5. The PTEN/AKT Pathway Participates in the Regulation of Pol-III-Directed Transcription Mediated by EGR1

**Figure 6:**
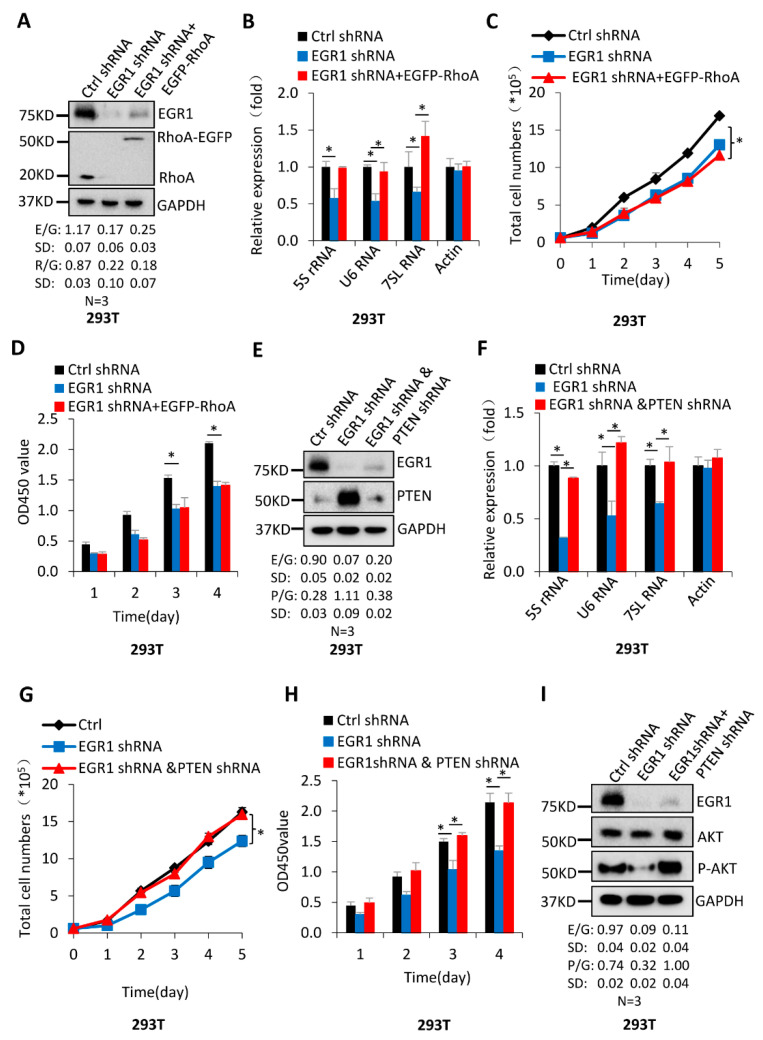
EGR1 regulates Pol-III-directed transcription by affecting PTEN/AKT signalling activity: (**A**) Western blotting was used to verify the generation of 293T cell line stably expressing both EGR1 shRNA and RhoA–EGFP; (**B**) RhoA–EGFP expression reversed the inhibition of U6 RNA and 7SL RNA expression caused by EGR1 downregulation. Pol III products were analysed using RT-qPCR using the cell lines generated in (**A**); (**C**,**D**) RhoA–EGFP expression did not change the inhibition of cell proliferation caused by EGR1 depletion. Cell counting (**C**) and MTT (**D**) assays were performed using the cell line generated in (**A**); (**E**) Western blot results showing the generation of the 293T cell line stably expressing both EGR1 shRNA and PTEN shRNA; (**F**) PTEN silencing reversed the inhibition of Pol III products expression caused by EGR1 downregulation. Pol III products were monitored with RT-qPCR using 293T cell lines generated in (**E**); (**G**,**H**) PTEN depletion alleviated the inhibition of cell proliferation caused by EGR1 downregulation. Cell counting (**G**) and MTT (**H**) assays were performed using the cell lines generated in (**E**); (**I**) EGR1 expression positively correlated with AKT phosphorylation levels in 293T cells. Western blotting was performed using the cell lines and the antibodies against the factors, as indicated. E/G, P/G, R/G, and pA/G, respectively represent the ratio of EGR1 (E), PTEN (P), RhoA (R), and p-AKT (pA) intensities to the GAPDH (G) intensity (*n* = 3). Each column in the point–line and bar graphs represents the mean ± SD of three biological replicates (*n* = 3). *, *p* < 0.05. *p* values for (**C**,**D**,**G**,**H**) were obtained by two-way ANOVA; *p* values for (**B**,**F**) were obtained by one-way ANOVA, followed by Bonferroni test, using a comparison of two groups among multiple groups.

RhoA is an effector upstream of an ERK signalling pathway that has been identified to regulate Pol-III-directed transcription [21]. To determine whether RhoA participates in the regulation of Pol-III-directed transcription mediated by EGR1, we generated 293T and HepG2 cell lines stably expressing both EGR1 shRNA and RhoA–EGFP, using a lentiviral infection system. Western blotting confirmed the generation of these cell lines was successful (Figure 6A and Figure S6A). Analysis of Pol III products showed that RhoA–EGFP expression could reverse the inhibition of U6 RNA and 7SL RNA expression caused by EGR1 downregulation but did not significantly change the inhibition of 5S rRNA expression induced by EGR1 depletion (Figure 6B and Figure S6B). Unexpectedly, RhoA–EGFP expression could not affect the repression of cell proliferation caused by EGR1 depletion (Figure 6C,D and Figure S6C,D), suggesting that RhoA contributed little to the inhibition of cell proliferation caused by EGR silencing. To determine whether PTEN mediated the repression of Pol-III-directed transcription caused by EGR1 silencing, we generated 293T and HepG2 cell lines stably expressing both EGR1 shRNA and PTEN shRNA using a lentiviral system and the cell line expressing EGR1 shRNA only, where the lentiviral vectors expressing EGR1 shRNA or PTEN shRNA carried with different fluorescent markers. Western blot data confirmed these cell lines were achieved successfully (Figure 6E and Figure S6E). Strikingly, PTEN silencing could reverse the inhibition of Pol III product expression caused by EGR1 silencing (Figure 6F and Figure S6F), and PTEN depletion could also abolish the repression of cell proliferation caused by EGR1 downregulation (Figure 6G,H and Figure S6G,H). These data suggest that PTEN participates in the regulation of Pol-III–directed transcription and cell proliferation mediated by EGR1. PTEN usually functions by affecting the activity of its downstream effector AKT [33]. To confirm if EGR1 regulated the AKT activity, we analysed AKT expression and phosphorylation in 293T and HepG2 cell lines with alteration of EGR expression by Western blotting. We found that EGR1 expression positively correlated with AKT phosphorylation levels. However, AKT expression was not affected by alteration of EGR1 expression (Figure S7A–D). Further assays showed that EGR1 downregulation reduced AKT phosphorylation, whereas PTEN silencing in the EGR1-depleted cells caused the increase in AKT phosphorylation (Figure 6I and Figure S7E). Thus, PTEN expression is inversely correlated with AKT phosphorylation levels (Figure 6E,I). PTEN has previously been reported to repress Pol-III-mediated transcription by targeting TFIIIB [33]. Our data indicate that the PTEN/AKT pathway links EGR1 and Pol-III-directed transcription and is required for the regulation of Pol-III-directed transcription mediated by EGR1.

### 2.6. EGR1 Is Required for the Inhibition of Pol-III-Directed Transcription Mediated FLNA

**Figure 7:**
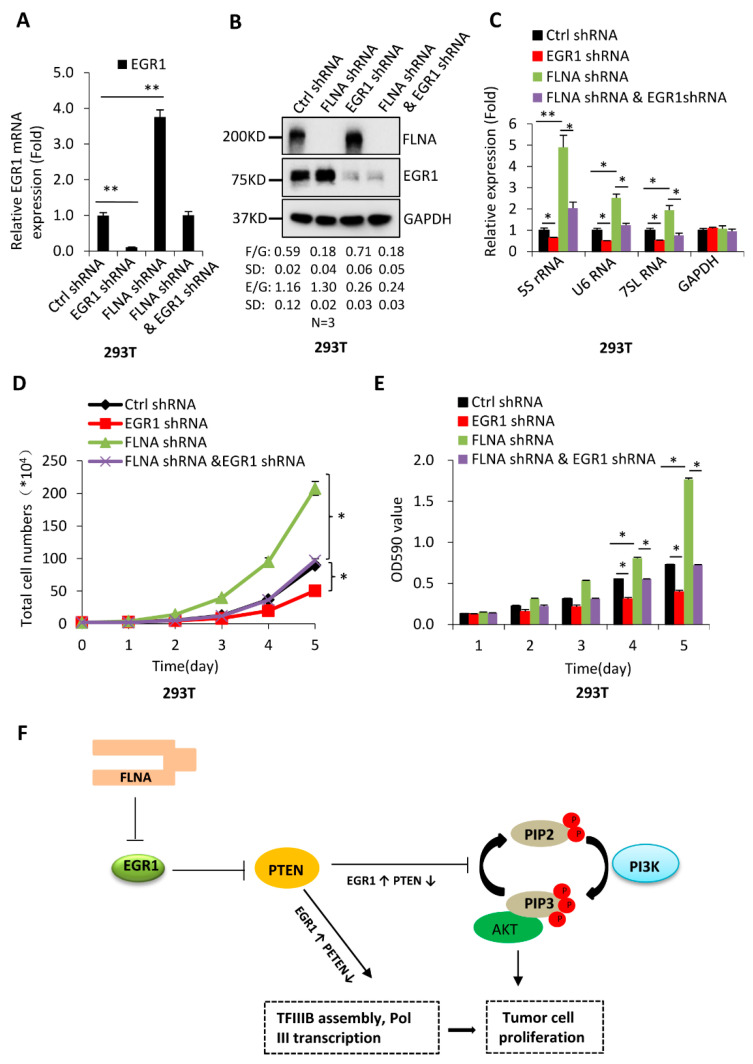
EGR1 is required for the regulation of Pol-III-directed transcription mediated by FLNA: (**A**,**B**) FLNA and EGR1 expression was determined by RT-qPCR (**A**) and Western blot (**B**) using 293T cell lines expressing both FLNA shRNA and EGR1 shRNA or FLNA shRNA only and the control cell line. F/G and E/G, respectively, represent the ratio of FLNA (F), and EGR1 (E) intensities to the GAPDH (G) intensity (*n* = 3); (**C**) EGR1 downregulation inhibited the activation of Pol-III-directed transcription caused by FLNA depletion in 293T cells. Expression of Pol III products was monitored by RT-qPCR using the cell line established in (**A**); (**D**,**E**) EGR1 downregulation inhibited the enhancement of cell proliferation caused by FLNA silencing. Cell proliferation activity was measured by cell counting (**D**) and MTT assays (**E**); (**F**) a pathway by which FLNA and EGR1 regulate Pol-III-directed transcription was proposed based on the data obtained in this study. Each column in the point–line and bar graphs represents the mean ± SD of three biological replicates (*n* = 3). *, *p* < 0.05; **, *p* < 0.01. *p* values for (**A**) were obtained by Student’s *t*-test by comparing two groups as indicated; *p* values for (**D**,**E**) were obtained by two-way ANOVA; *p* values for (**C**) were obtained by one-way ANOVA, followed by Bonferroni test, with a comparison of two groups among multiple groups.

Our previous research has shown that FLNA silencing activates Pol-III-directed transcription [38]. As demonstrated above, FLNA downregulation activated EGR1 expression (Figure S1). Whether EGR1 participates in the regulation of Pol-III-directed transcription mediated by FLNA remains unclear. To address this question, we generated 293T and HeLa cell lines stably expressing both FLNA shRNA and EGR1 shRNA using the cell line expressing FLNA shRNA and a lentiviral infection system. Both RT-qPCR and Western blot data confirmed that the cell lines were successfully achieved (Figure 7A,B and Figure S8A,B). Analysis of Pol III products showed that FLNA depletion stimulated the expression of Pol III products. However, EGR1 silencing impeded the activation of Pol-III-directed transcription caused by FLNA downregulation (Figure 7C and Figure S8C). Cell proliferation assays showed that FLNA depletion promoted proliferation activity of 293T and HeLa cell lines, whereas the increased cell proliferation was suppressed when EGR1 was down-regulated in the FLNA-depleted cell lines (Figure 7D,E and Figure S8D,E). These data indicate that EGR1 is also required for the inhibition of Pol-III-directed transcription mediated by FLNA.

Based on the data obtained in this study, we proposed a pathway by which FLNA and EGR1 regulate Pol-III-directed transcription. Specifically, FLNA depletion stimulates EGR1 expression, which subsequently inhibites PTEN expression. PTEN inhibition activates AKT phosphorylation, which induces activation of Pol-III-directed transcription and cell growth (Figure 7F).

## 3. Discussion

Pol-III-directed transcription can be strictly regulated by oncogenic factors, tumour suppressors, signalling factors, etc. [21,26]. However, the regulatory network governing Pol-III-dependent transcription remains to be clarified. In this study, we found that transcription factor EGR1, a GC-rich sequence-binding factor, can positively regulate Pol-III-directed transcription (Figure 1, Figure 2 and Figure 3). EGR1 has been found to regulate the expression of diversified target genes, which controls several important biological processes, including cell cycle and proliferation, cell invasion and migration, and angiogenesis [1,2,3]. However, these target genes are protein-coding genes that are transcribed by RNA polymerase II. Thus, we identified a novel role of EGR1 in transcription regulation in this study.

Alteration of EGR1 expression did not change TBP, BRF1, and GTF3C2 expression in 293T, HeLa, and HepG2 cells (Figure 5E,F and Figure S5A–D); this suggests that EGR1 indirectly modulates expression of Pol III products. It has been shown that EGR1 directly regulates p53 expression by binding their promoters [3]. Tumour suppressor p53 has been confirmed to inhibit Pol-III-directed transcription by targeting the general transcription factor TFIIIB [39]. Unexpectedly, the expression of p53 and MDM2 was not affected by EGR1 silencing or overexpression in the cells we investigated (Figure 5G,H and Figure S5E,F). This result is inconsistent with the previous findings, according to which EGR1 positively regulates p53 expression [3,4,40]. The expression of oncogenic c-MYC that indirectly regulates Pol-III-directed transcription [26] remained unchanged after EGR1 depletion or overexpression (Figure 5G,H and Figure S5E,F). It has been shown that EGR1 positively regulates PTEN expression by binding PTEN promoters [3,41]. In this study, however, we found that EGR1 depletion stimulated PTEN expression, whereas EGR1 overexpression inhibited PTEN expressions. This result is distinct from the previous finding [3,41]. The G-protein RhoA, an upstream signalling factor, mainly mediates cell movement or migration and cytoskeleton maintenance, although it is required for other cellular activities [42]. In this study, we found that EGR1 expression positively correlated with RhoA expression levels. These data suggest that either PTEN or RhoA are required for the regulation of Pol III transcription mediated by EGR1. In rescue experiments, RhoA–EGFP expression could reverse the expression inhibition of some Pol III products caused by EGR1 silencing, but it could not rescue the inhibition of cell proliferation caused by EGR1 downregulation. In contrast, PTEN downregulation in the EGR1-depleted cell lines reversed the inhibition of Pol-III-mediated transcription and cell proliferation caused by EGR1 (Figure 6A–H and Figure S6A–H). Furthermore, the increased expression of PTEN inhibited AKT phosphorylation (Figure 6I and Figure S6I). PTEN has been reported to inhibit Pol III transcription, where PTEN inhibits the association of TBP with BRF1 and leads to a reduction in Pol III transcription assembly at the tRNA–Leu gene [33]. Thus, our data suggest that EGR1 regulates Pol-III-directed transcription by affecting the PTEN/AKT signalling activity, although the roles of other possible pathways cannot be excluded in this process.

EGR1 was originally recognised as a tumour suppressor because EGR1 positively regulates TGF-β 1, p53, and PTEN expression to induce cell apoptosis [3]. In this study, however, we found that EGR1 can promote the proliferation activity of several transformed cell lines, including 293T, HeLa, and HepG2 cells, suggesting that EGR1 plays a positive role in the proliferation of these cell lines. Additionally, Pol-III-specific inhibitor assays indicate that EGR1 may promote cell proliferation by activating Pol-III-directed transcription (Figure 4 and Figures S2–S4). EGR1 has been reported to promote cell proliferation in prostate, lung, and gastric cancers. Thus, our results obtained in this research are consistent with these findings [1,2]. Since EGR1 plays a positive role in cancer cell development, EGR1 can be applied to anticancer research in the future. Collectively, in this study, we identified a novel role of EGR1 in Pol-III-directed transcription and cell proliferation in transformed cell lines. Our findings provide novel insights into the regulatory mechanism of Pol-III-directed transcription and the mechanism of cell proliferation mediated by EGR1.

## 4. Materials and Methods

### 4.1. Plasmids, Cells and Reagents

The lentiviral expression vectors, including pLV-U6-mCherry-Puro (Cat No. VL3104), pLV-U6-EGFP-Puro (Cat No. VL3103), and pLV-EF1α-EGFP-Puro (Cat No. VL3311), were purchased from Inovogen Tech Co (Beijing, China). FLNA shRNA lentiviral particles (Cat No. sc-35374-V) and control shRNA lentiviral particles (Cat No. sc-108080) were purchased from Santa Cruz Biotech. Cell types, including SaOS2 (HTB-85, ATCC), 293T (CRL-3216, ATCC), HeLa (CCL-2, ATCC), and HepG2 (HB-8065, ATCC), were obtained from the American Type Culture Collection (ATCC) and cultured in their respective media supplied with 10% foetal bovine serum (Cat No. 10100, Gibco) and 1× penicillin–streptomycin (Cat No. 15140122, Gibco). DNA and RNA oligonucleotides were ordered from Sangon (Shanghai, China). DNA purification kits (Cat No. AP-GX-250) were purchased from Corning Co. Enzymes and transfection reagents (Cat No. 11668019) were derived from Thermo Fisher Scientific. All chemicals were obtained from Merk Co.

### 4.2. Gene Cloning

EGR1 shRNA-coding DNA fragments were synthesised and cloned downstream of the U6 promoter at the pLV-U6-mCherry-Puro plasmid. PTEN shRNA-coding DNA fragments were cloned downstream of the U6 promoter at the pLV-U6-EGFP-Puro plasmid. An *HA–EGR1* fusion gene was cloned downstream of the EF1α promoter at the pLV-EF1α-EGFP-Puro. A RhoA gene was amplified from the cDNA sample, which was synthesised using the 0.5 μg RNA extracted from 293T cells and 5 units of reverse transcriptase (Cat No. 28025013, Thermo Scientific, USA). The resulting Rho A DNA fragments were cloned downstream of the EF1α promoter at the pLV-EF1α-EGFP-Puro.

### 4.3. Transfection and Generation of Stable Cell Lines

For EGR1 siRNA transient transfection, 293T and HeLa cells (3 × 10^5^ cells for each) were inoculated and cultured in 12-well plates for 24 h. The cells in each well were transfected with a 200 μL transfection medium containing 6 pmol of siRNA fragments and 2 μL of TuborFect (Cat. No. R0531), according to the manual provided by Thermo Scientific. After 48 h, cells were harvested and subjected to analysis of EGR1 expression using RT-qPCR and Western blot techniques. For the generation of the cell lines stably expressing shRNA or protein, 6 × 10^6^ 293T cells were cultured for 24 h in 6-well plates before transfection. Transient transfection was performed using a 400 μL transfection medium containing 3 μg of protein- or shRNA-expressing lentiviral vectors and 3 μg of the packaging vectors pH1 and pH2 (Cat No. KLV3501, Inovogen, Beijing, China). After 48 h, the culture medium containing lentiviral particles was collected and filtrated using a sterile filter with a 0.45 μm pore size. The resulting medium (0.5 mL for each cell type) was used to infect 293T, HeLa, and HepG2 cells cultured on 12-well plates. The cells infected were selected for 72 h using the complete medium containing 10 μg/mL puromycin and then screened by diluting the cells into 96-well plates. The drug-resistant cell colonies were picked from the 96-well plates and cultured in 12-well plates. The cell lines stably expressing shRNA or fusion protein were verified by RT-qPCR and Western blotting. For the generation of the SaOS2 cell line stably expressing FLNA shRNA, SaOS2 cells grown in 12-well plates were directly infected with lentiviral particles, the remaining procedures followed the protocol as described above. For the generation of the cell lines stably expressing both FLNA shRNA and EGR1 shRNA, the FLNA shRNA-expressing cell line was further infected with the lentiviral particles expressing EGR1 shRNA. After selection, the positive cell lines expressing both FLNA shRNA and EGR1 shRNA were determined by Western blotting using the antibodies against FLNA or EGR1. For other combined cell lines, including EGR1 shRNA versus PTEN shRNA or EGR1 shRNA versus RhoA–EGFP, The procedures were followed as described in the generation of the cell line stably expressing FLNA shRNA and EGR1 shRNA.

### 4.4. RT-qPCR and Detection of Pol III Products

Briefly, 293T cells or other cell types were seeded in 12-well plates. At 90% confluence, cells were harvested and total RNA was extracted using an RNA extraction kit (Cat No. 74004, QIAGEN, Germany). An amount of 0.5 μg RNA was used for cDNA synthesis at 42 °C in the 20 μL reaction mixture containing 5 units of reverse transcriptase. After incubating 1 h, the reaction mixture was diluted 5 times with ddH_2_O, and 1 μL cDNA was used for quantitative PCR (qPCR) in which 20 μL reaction mixtures contained 10 μL 2× SYBR Green Master Mix (Cat No. 1725271, Bio-Rad, USA) and 5 pmoles of the primers for detection of Pol III products. Quantitative PCR reactions were performed using the Bio-Rad Real-Time PCR Detection System. The data were analysed with the CFX Manager 3.0 software (Bio-Rad, USA), where the *Actin* gene was used as a reference gene.

### 4.5. Western Blot Analysis

For this analysis, 293T, HeLa, and HepG2 cells were grown and cultured in 6-well plates. At 90% confluence, cells were harvested and analysed with 250 μL 1× SDS loading buffer. After incubating for 10 min at 100 °C, Western blot assays were performed using 20 μL cell lysate and the antibodies against the factors, including EGR1 (Ab194357, Abcam, UK), HA(H9658; Sigma-Aldrich, USA), GAPDH (RAB0101, Frdbrio, Wuhan, China), BRF1 (SC-81405, Santa Cruz Biotech, USA.), GTF3C2 (SC-81406, Santa Cruz Biotech, USA), c-MYC (SC-40, Santa Cruz Biotech, USA), MDM2 (SC-965, Santa Cruz Biotech, USA), p53 (SC-126, Santa Cruz Biotech, USA), RhoA (SC-418, Santa Cruz Biotech, USA), PTEN (SC-7974, Santa Cruz Biotech, USA), and EGFP (TAG0070, FrdBio, Wuhan China).

### 4.6. Cell Proliferation Assays

Cell proliferation assays were performed using several different methods, including cell counting, MTT, CCK8, and EdU. For cell counting and EdU assays, the cell lines with EGR1 depletion or overexpression and their control cell lines were seeded in 12-well plates. The cell counting assays were performed as described previously [43], while EdU assays followed the procedures as described in a recent paper [44]. For MTT assays, the cell lines with EGR1 depletion or overexpression and their control cell lines were grown in 96-well plates. The MTT assays were performed as described previously [43]. For CCK8 assays, the cell lines stably expressing both EGR1 shRNA and PTEN shRNA (or expressing both EGR1 shRNA and Rho–EGFP) and their control cell lines were cultured for 24 h in 96-well plates. Next, 10 μL of CCK-8 solution (Cat No. A311-01, Vazyme, Nanjing, China) was added to each well and cultured for 4 h at 37 °C. The absorbance (450 nm) for each well in the plates was measured using the SpectraMax i3 equipment (Molecular Device).

### 4.7. ChIP Assays

The 293T cell lines stably expressing GATA4 shRNA or control shRNA were grown in 10 cm dishes. At 90% confluence, the cell lines were fixed for 10 min using a 10 mL PBS solution containing 1% formaldehyde and then quenched with 1 mL glycine solution (2.5 M). After washing three times with 1× PBS solution, the cells were suspended with 1 mL ChIP lysis buffer (1% SDS, 10 mM EDTA, 50 mM Tris-HCl, pH 8.0) and disrupted with an ultrasonicator. After centrifugation at 12,000 rpm, the supernatant was retained and diluted 6 times with a ChIP dilution buffer (0.01% SDS, 1.1% TritonX-100, 1.2 mM EDTA, 16.7 mM Tris-HCl PH 8.0 and 167 mM NaCl). The rest procedures for ChIP assays followed, as described previously [43]. ChIP assays were performed using the antibody against BRF1 (SC-81405, Santa Cruz Biotech.), GTF3C2 (SC-81406, Santa Cruz Biotech.), and POLR3A (SC-292119, Santa Cruz Biotech.). After elution, the chromatin samples immunoprecipitated were subjected to de-crosslinking and DNA purification. The DNA from each ChIP sample was eluted with 40 μL ddH_2_O. One μL of them (1/40) for individual factors was used for a qPCR reaction; meanwhile, 0.01% input DNA was used as a positive control. Relative occupancy was obtained by comparing the relative quantity of promoter DNA in 1 μL of ChIP sample to that in 0.01% input DNA.

### 4.8. Statistical Analysis

The experiments of gene expression, cell proliferation, and ChIP assays were performed in three biological replicates. The mean and standard deviation (SD) for the data from gene expression detection, cell proliferation assays, and ChIP-qPCR were obtained with the GraphPad Prism 6.0 software. Each column or point in the graphs represents the mean ± SD (*n* = 3). *p* values for the gene expression comparison between control and treatment groups were obtained by Student’s *t*-test; *p* values for cell proliferation comparison of multiple groups were obtained by two-way ANOVA. *p* values for the gene expression comparison between multiple groups were obtained by one-way ANOVA, followed by Bonferroni test, using a comparison of two groups within multiple groups.

## Data Availability

The publicly achived data used in this study were obtained from the NCBI repository online (https://www.ncbi.nlm.nih.gov/Traces/study/?acc=PRJNA726417, Submission ID: SUB9541028, accessed on 4 May 2021).

## Supplementary Materials

The following supporting information can be downloaded at: https://www.mdpi.com/article/10.3390/ijms23094930/s1.
