# Supplementary material for: Early Growth Response 1 Strengthens Pol-III-Directed Transcription and Transformed Cell Proliferation by Controlling PTEN/AKT Signalling Activity
Source: Int J Mol Sci. 2022 Apr 29;23(9):4930. doi: 10.3390/ijms23094930 (PMC9105817; doi:10.3390/ijms23094930)
Supplement: Supplementary file 1 [file ijms-23-04930-s001.zip › ijms-1557396-supplementary.pdf]

Supporting information

Early growth response 1 strengthens Pol III-directed transcription and transformed cell proliferation by controlling PTEN/AKT signalling activity

Zhongyu Wu[1#], Liyun Huang[1#], Shasha Zhao[1], Juan Wang[1,2], Cheng Zhang[1], Xiaoye Song[1], Qiyue Chen[1], Jiannan Du[1], Deen Yu[1], Xiaomeng Sun[1], Yue Zhang[1], Shihua Zhang[1], Wensheng Deng[1*], Huan Deng[1*]

1. School of Life Science and Health, Wuhan University of Science and Technology

2. School of Materials and Metallurgy, Wuhan University of Science and Technology

#. These authors contributed this work equally.

*. Correspondence, dengwensheng@wust.edu.cn, denghuan@wust.edu.cn

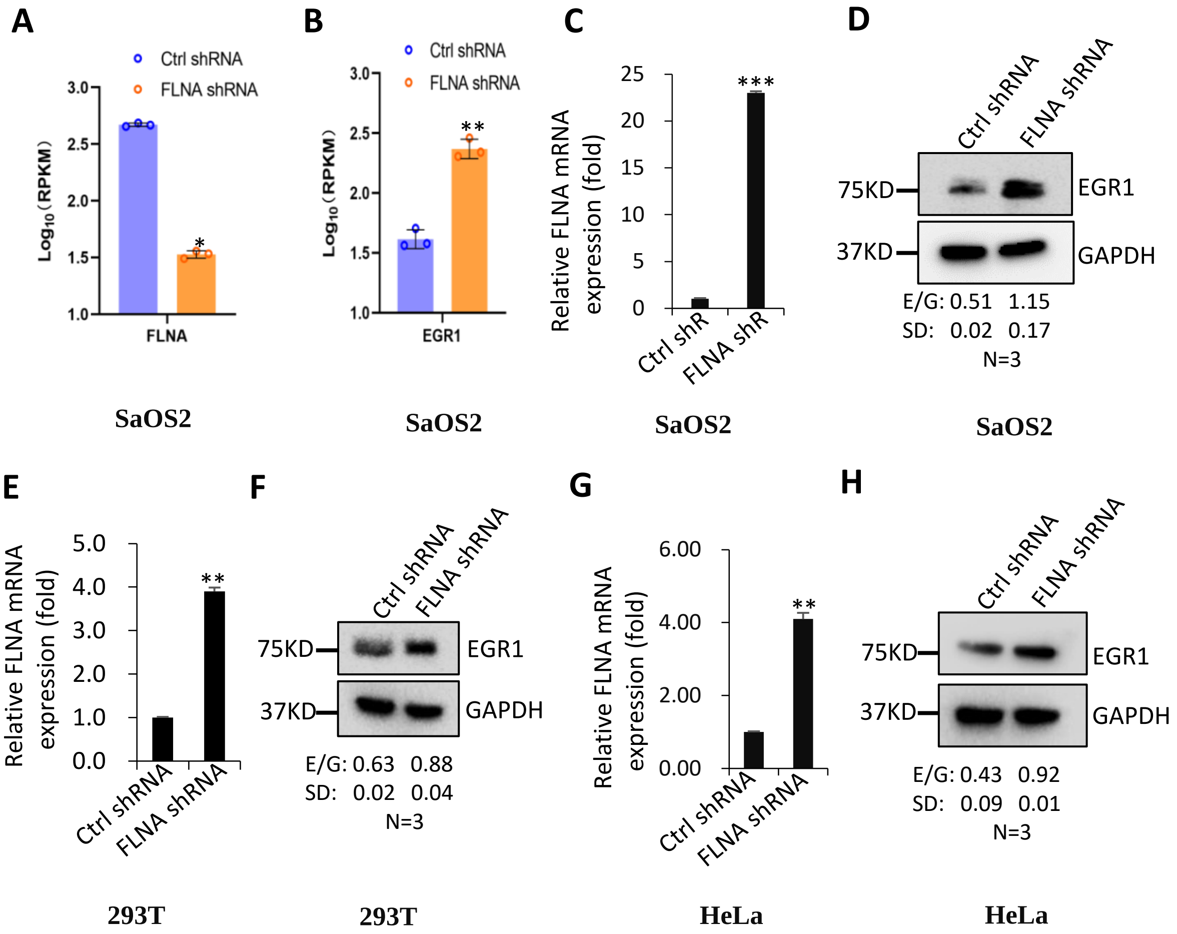

Figure S1. FLNA silence stimulated EGR1 expression. (A) and (B) RNA-seq data showing the effect of FLNA silence on FLNA (A) and EGR1 (B) mRNA expression. (C, D) Expression of EGR1 was detected by RT-qPCR (C) and Western blot (D) in SaOS2 cell lines stably expressing FLNA shRNA or control shRNA. E/G represents the ratio of the EGR1 intensity to the GAPDH intensity (n=3). . SD, Standard deviation. (F-H) Expression of EGR1 was detected by RT-qPCR (F) and Western blot (G) in 293T cell lines stably expressing FLNA shRNA or control shRNA. H is the quantification result of Western blot data (n=3). (I-k) Expression of EGR1 was detected by RT-qPCR (I) and Western blot (J) in HeLa cell lines stably expressing FLNA shRNA or control shRNA. E/G in D, F and H represents the ratio of the EGR1 (E) intensity to the GAPDH (G) intensity (n=3); SD, Standard deviation. Each column in A, B, C, E and H represents the mean ± SD of three biological replicates (n=3). *, $p < 0.05$; **, $p < 0.01$. *P* values were obtained by a student's *t*-test performed with control and treatment groups.

Fig. S2

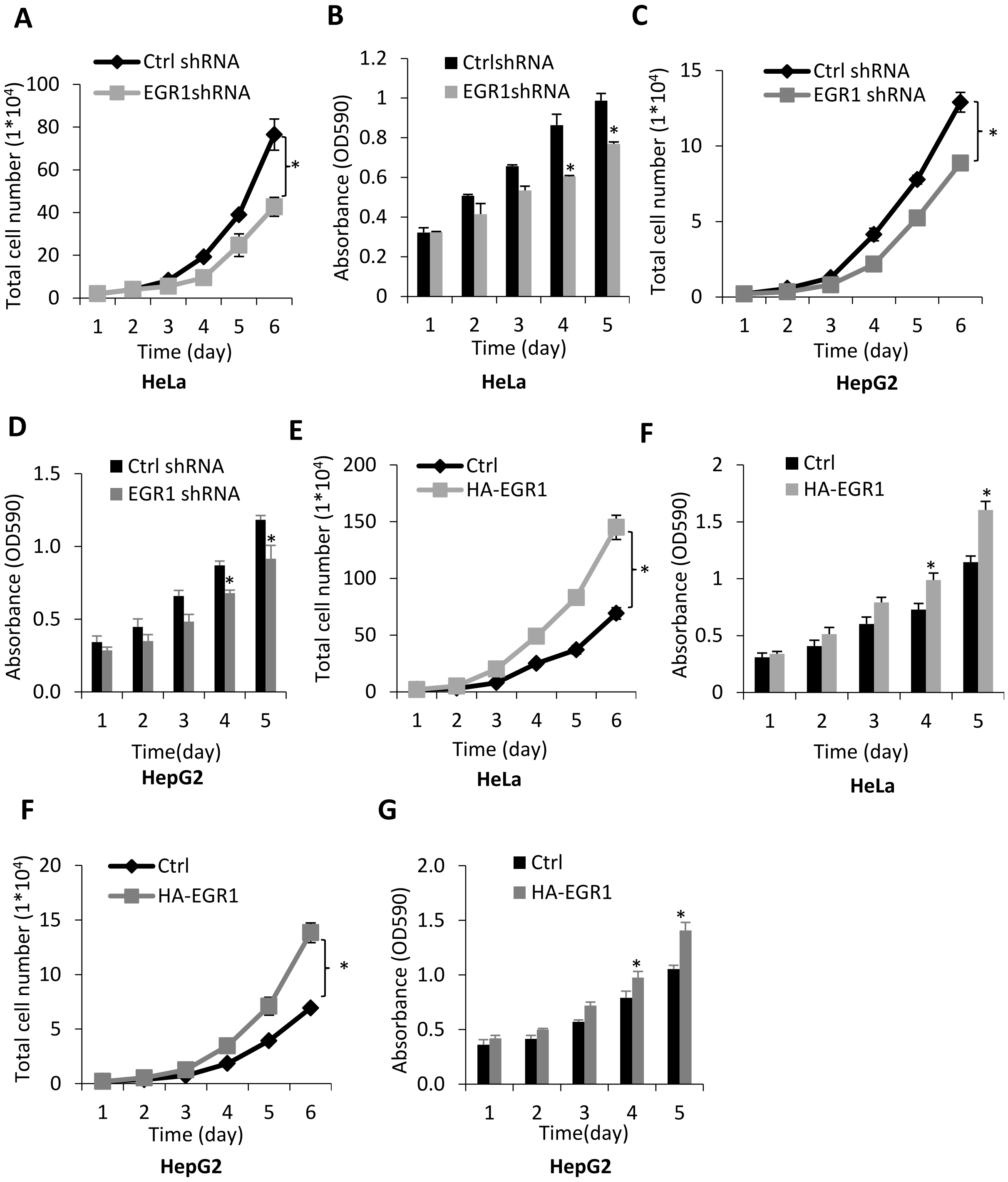
A
Ctrl shRNA
EGR1shRNA
Total cell number ($1*10^4$)
Time (day)
HeLa
B
CtrlshRNA
EGR1shRNA
Absorbance (OD590)
Time (day)
HeLa
C
Ctrl shRNA
EGR1 shRNA
Total cell number ($1*10^4$)
Time (day)
HepG2
D
Ctrl shRNA
EGR1 shRNA
Absorbance (OD590)
Time(day)
HepG2
E
Ctrl
HA-EGR1
Total cell number ($1*10^4$)
Time (day)
HeLa
F
Ctrl
HA-EGR1
Absorbance (OD590)
Time (day)
HeLa
F
Ctrl
HA-EGR1
Total cell number ($1*10^4$)
Time (day)
HepG2
G
Ctrl
HA-EGR1
Absorbance (OD590)
Time(day)
HepG2

Figure S2. EGR1 promotes HeLa and HepG2 cell proliferation. (A) and (B) EGR1 knockdown inhibited HeLa cell proliferation. A HeLa cell line stably expressing EGR1 shRNA and its control cell line were cultured in 12-well and 96-well plates, cell proliferation activity was examined by cell counting (A) and MTT assays (B), respectively. (C) and (D) EGR1 silence reduced HepG2 cell proliferation activity. Cell counting (C) and MTT assays (D) were performed using HepG2 cell lines stably expressing EGR1 shRNA and its control cell line. (E) and (F) EGR1 overexpression stimulated HeLa cell proliferation. A HeLa cell line stably expressing HA-EGR1 and its control cell line were cultured in 12-well and 96-well plates, cell proliferation activity was examined by cell counting (E) and MTT assays (F). (G) and (H) EGR1 overexpression promoted HepG2 cell proliferation. Cell counting (E) and MTT assays (F) were performed using a HepG2 cell line stably expressing HA-EGR1 and its control cell line. Each column in the bar graphs represents the mean $\pm$SD of three biological replicates (n=3). *, $p < 0.05$; **, $p < 0.01$. *P* values were obtained by two-way ANOVA.

Fig. S3

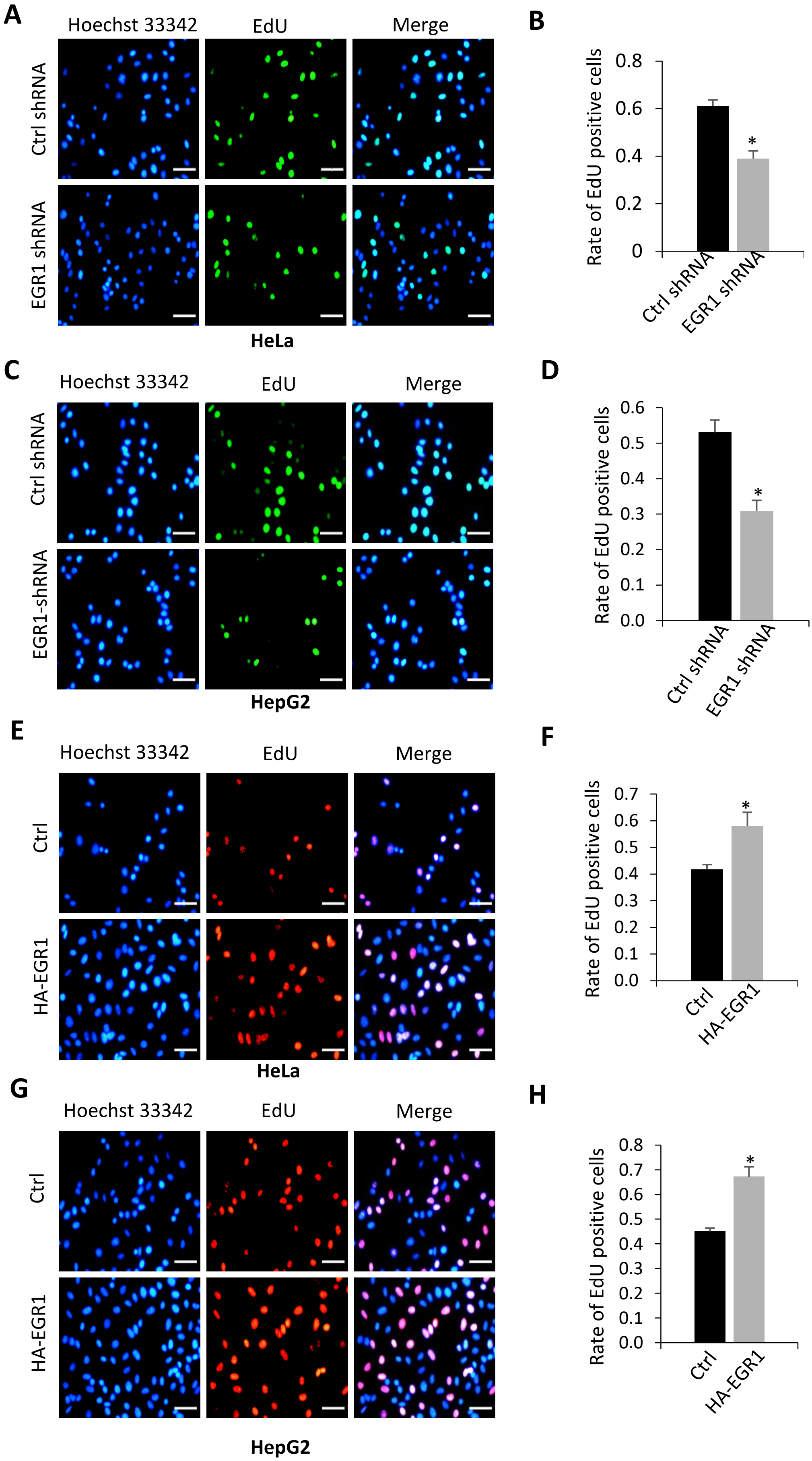
A
Hoechst 33342
EdU
Merge
Ctrl shRNA
EGR1 shRNA
HeLa
B
Rate of EdU positive cells
0.8
0.6
0.4
0.2
0
*
Ctrl shRNA
EGR1 shRNA
C
Hoechst 33342
EdU
Merge
Ctrl shRNA
EGR1-shRNA
HepG2
D
Rate of EdU positive cells
0.6
0.5
0.4
0.3
0.2
0.1
0.0
*
Ctrl shRNA
EGR1 shRNA
E
Hoechst 33342
EdU
Merge
Ctrl
HA-EGR1
HeLa
F
Rate of EdU positive cells
0.7
0.6
0.5
0.4
0.3
0.2
0.1
0.0
*
Ctrl
HA-EGR1
G
Hoechst 33342
EdU
Merge
Ctrl
HA-EGR1
HepG2
H
Rate of EdU positive cells
0.8
0.7
0.6
0.5
0.4
0.3
0.2
0.1
0.0
*
Ctrl
HA-EGR1

Figure S3. EdU assays verified the positive role of EGR1 in HeLa and HepG2 cell proliferation (A-D) EdU assay results showing the effect of EGR1 depletion on HeLa and HepG2 cell proliferation activity. EdU assays were performed using HeLa (A, B) and HepG2 (C, D) cell lines stably expressing EGR1 shRNA or control shRNA. Cell samples were imaged under a fluorescent microscope with a 10x objective lens (A, C). The rate of EdU positive cells was obtained by comparing the number of EdU-labelled cells to that of total cells within minimal 5 images randomly selected (B, D). (E-H) EdU assay results showing the effect of EGR1 overexpression on HeLa and HepG2 cell proliferation activity. EdU assays were performed using HeLa (E, F) and HepG2 (G, H) cell lines stably expressing HA-EGR1 and their control cell lines. Cell samples were imaged under a fluorescent microscope with a 10x objective lens (E, G). The rate of EdU positive cells was obtained for B and D (F, H). The scale bars in the images represent 50 µm. *, Each column in the bar graphs represents the mean $\pm$ SD of three biological replicates (n=3). $p < 0.05$; P values were obtained by a student's t-test performed with control and treatment groups.

Fig. S4

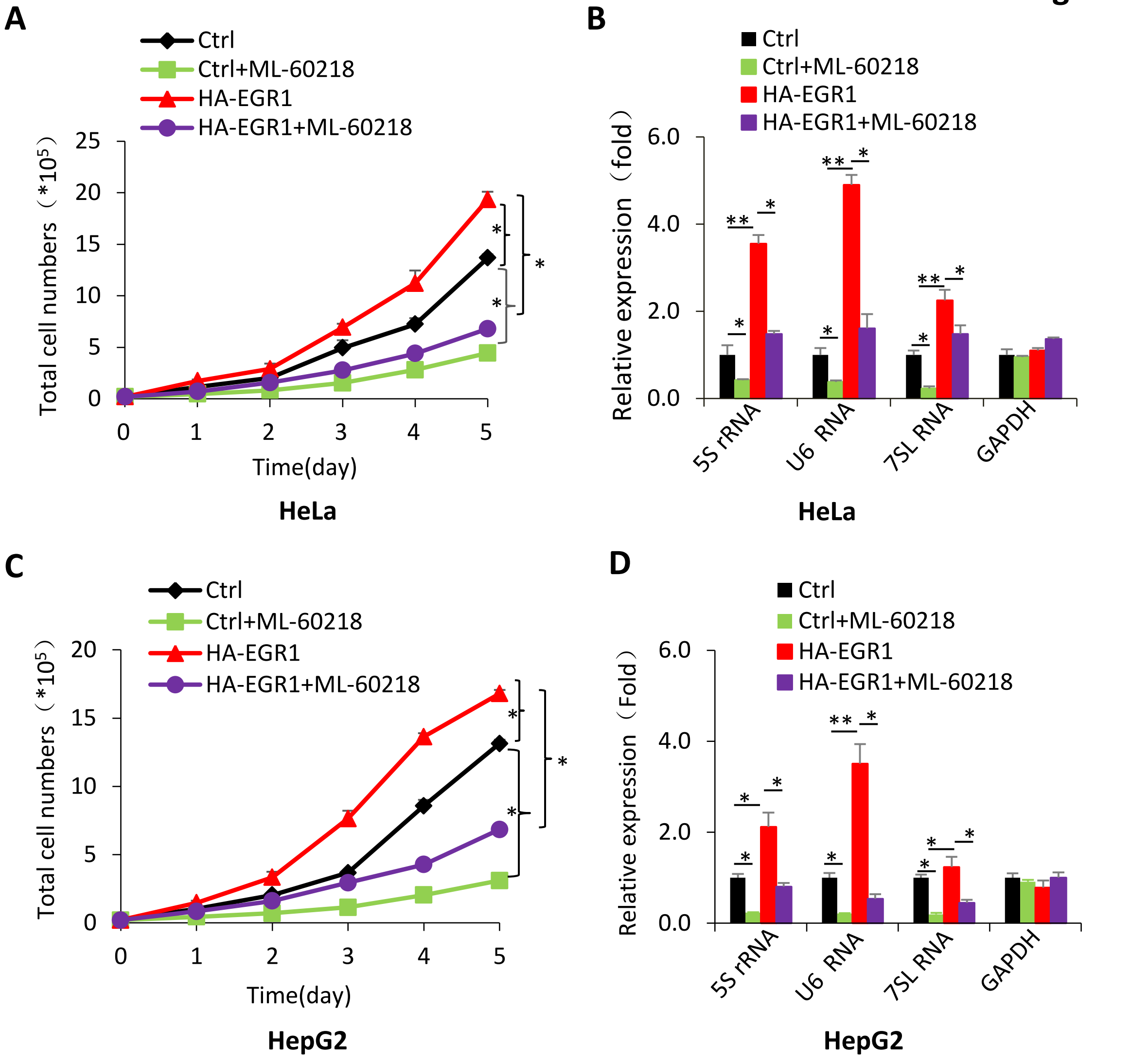

Figure S4. ML-60218 inhibited both the enhancement of cell proliferation and the activation of Pol III-directed transcription caused by EGR1 overexpression (A) The presence of ML-60218 reduced the enhancement of HeLa cell proliferation induced by EGR1 overexpression . (B) The presence of ML-60218 inhibited the activation of Pol III-directed transcription caused by EGR1 overexpression in HeLa cells. (C) The inhibitor ML-60218 impeded the increase of HepG2 cell proliferation induced by EGR1 overexpression . (D) The inhibitor ML-60218 inhibited activation of Pol III-directed transcription caused by EGR1 overexpression in HepG2 cells . *, $p < 0.05$; **, $p < 0.01$. Each column or point in graphs represents the mean ± SD of three biological replicates (n=3). *P* values for A and C were obtained by two-way ANOVA; *p* values for B and D were obtained by one-way ANOVA, followed by Bonferroni test through comparing two groups within multiple groups.

Fig. S5

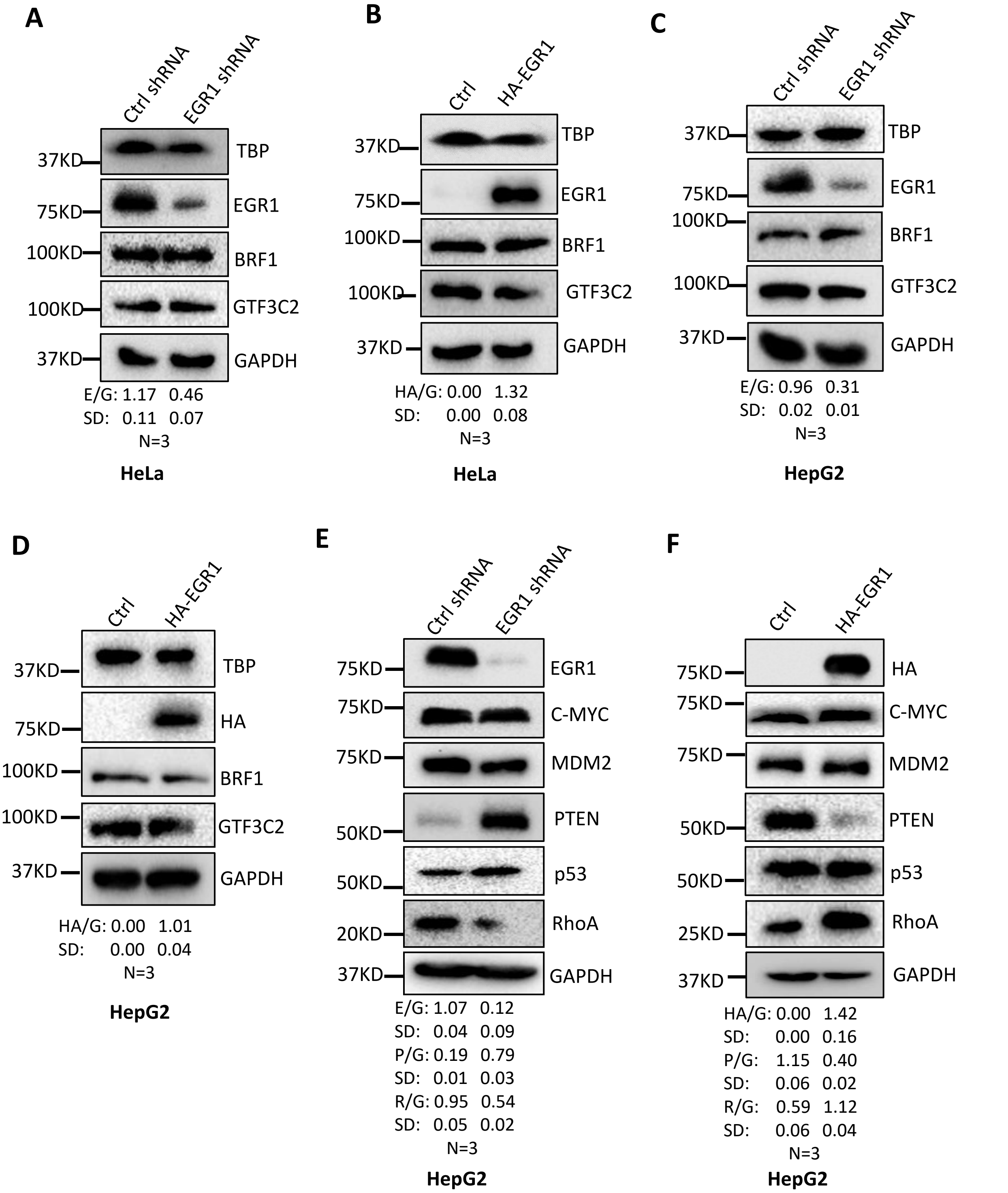
A
Ctrl shRNA
EGR1 shRNA
37KD
TBP
75KD
EGR1
100KD
BRF1
100KD
GTF3C2
37KD
GAPDH
E/G: 1.17 0.46
SD: 0.11 0.07
N=3
HeLa
B
Ctrl
HA-EGR1
37KD
TBP
75KD
EGR1
100KD
BRF1
100KD
GTF3C2
37KD
GAPDH
HA/G: 0.00 1.32
SD: 0.00 0.08
N=3
HeLa
C
Ctrl shRNA
EGR1 shRNA
37KD
TBP
75KD
EGR1
100KD
BRF1
100KD
GTF3C2
37KD
GAPDH
E/G: 0.96 0.31
SD: 0.02 0.01
N=3
HepG2
D
Ctrl
HA-EGR1
37KD
TBP
75KD
HA
100KD
BRF1
100KD
GTF3C2
37KD
GAPDH
HA/G: 0.00 1.01
SD: 0.00 0.04
N=3
HepG2
E
Ctrl shRNA
EGR1 shRNA
75KD
EGR1
75KD
C-MYC
75KD
MDM2
50KD
PTEN
50KD
p53
20KD
RhoA
37KD
GAPDH
E/G: 1.07 0.12
SD: 0.04 0.09
P/G: 0.19 0.79
SD: 0.01 0.03
R/G: 0.95 0.54
SD: 0.05 0.02
N=3
HepG2
F
Ctrl
HA-EGR1
75KD
HA
75KD
C-MYC
75KD
MDM2
50KD
PTEN
50KD
p53
25KD
RhoA
37KD
GAPDH
HA/G: 0.00 1.42
SD: 0.00 0.16
P/G: 1.15 0.40
SD: 0.06 0.02
R/G: 0.59 1.12
SD: 0.06 0.04
N=3
HepG2

Figure S5. EGR1 regulates expression of PTEN and RhoA . (A) and (B) Alteration of EGR1 expression did not affect expression of TBP, BRF1 and GTF3C2 in HeLa cells**.** Western blot was performed using HeLa cell lines expressing EGR1 shRNA (A) or HA-EGR1 (B) and the antibodies against the factors as indicated**.** (C) and (D) Alteration of EGR1 expression did not affect expression of TBP, BRF1 and GTF3C2 in HepG2 cells**.** Western blot was performed using HepG2 cell lines expressing EGR1 shRNA (A) or HA-EGR1 (B) and the antibodies against the factors as indicated. (E) The effect of EGR1 silence on expression of oncogenic factors, tumour repressors and signalling factors. Western blot was performed using a HepG2 cell line expressing EGR1 shRNA and its control cell line and the antibodies against the factors as indicated. (F) The effect of EGR1 overexpression on expression of oncogenic factors, tumour repressors and signalling factors. Western blot was performed using a HepG2 cell line expressing HA-EGR1 shRNA and its control cell line and the antibodies against the factors as indicated. E/G, H/G, P/G and R/G respectively represent the ratio of EGR1 (E), HA-EGR1(H), PTEN(P) and RhoA (R) intensities to the GAPDH (G) intensity (n=3).

Fig. S6

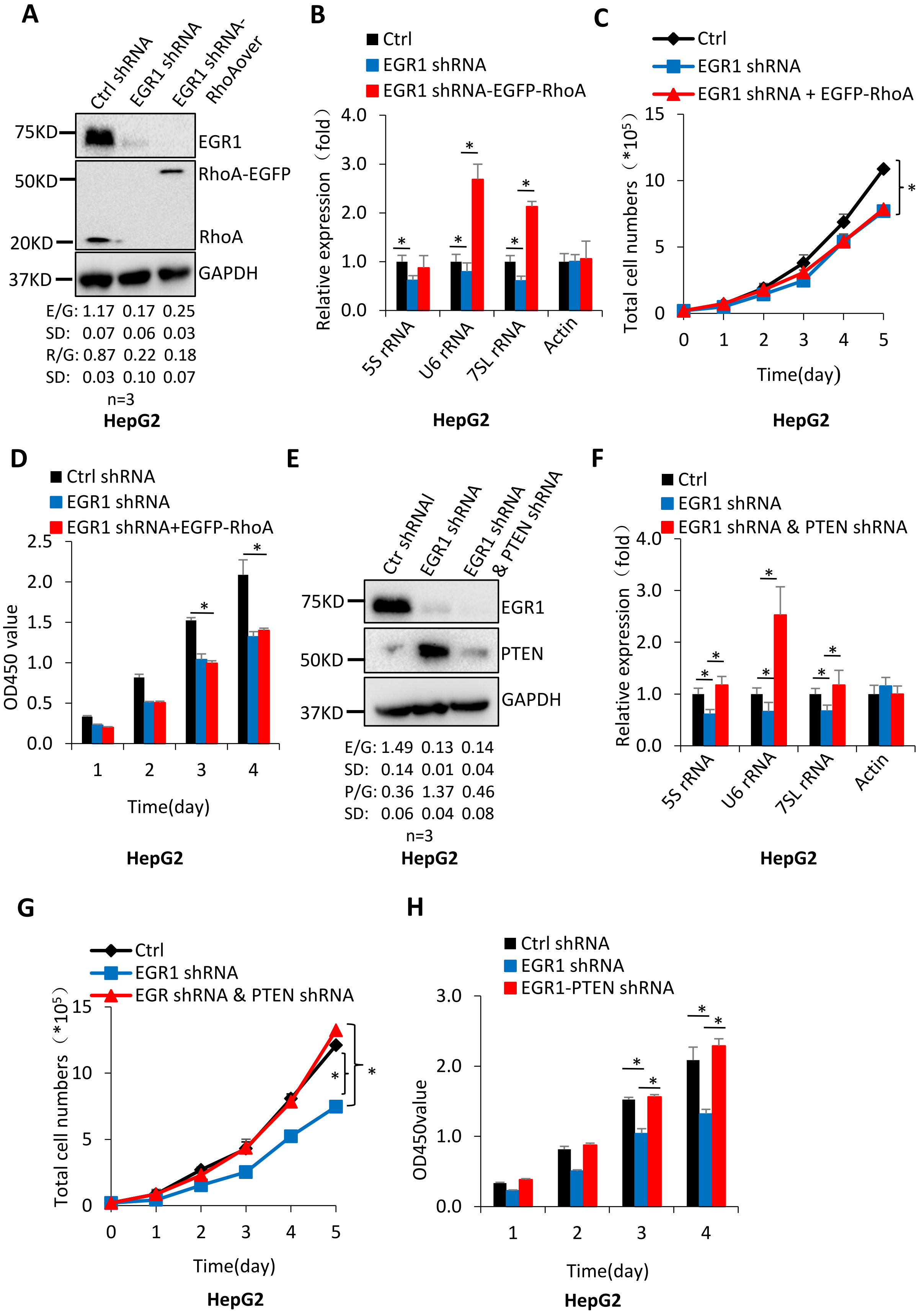
A
Ctrl shRNA
EGR1 shRNA
EGR1 shRNA-RhoAover
75KD
50KD
20KD
37KD
EGR1
RhoA-EGFP
RhoA
GAPDH
E/G: 1.17 0.17 0.25
SD: 0.07 0.06 0.03
R/G: 0.87 0.22 0.18
SD: 0.03 0.10 0.07
n=3
HepG2
B
Ctrl
EGR1 shRNA
EGR1 shRNA-EGFP-RhoA
Relative expression（fold）
4.0
3.0
2.0
1.0
0.0
*
5S rRNA
U6 rRNA
7SL rRNA
Actin
HepG2
C
Ctrl
EGR1 shRNA
EGR1 shRNA + EGFP-RhoA
Total cell numbers（*10^5）
15
10
5
0
0 1 2 3 4 5
Time(day)
*
HepG2
D
Ctrl shRNA
EGR1 shRNA
EGR1 shRNA+EGFP-RhoA
OD450 value
2.5
2.0
1.5
1.0
0.5
0.0
1 2 3 4
Time(day)
*
HepG2
E
Ctr shRNAI
EGR1 shRNA
EGR1 shRNA & PTEN shRNA
75KD
50KD
37KD
EGR1
PTEN
GAPDH
E/G: 1.49 0.13 0.14
SD: 0.14 0.01 0.04
P/G: 0.36 1.37 0.46
SD: 0.06 0.04 0.08
n=3
HepG2
F
Ctrl
EGR1 shRNA
EGR1 shRNA & PTEN shRNA
Relative expression（fold）
4.0
3.0
2.0
1.0
0.0
*
5S rRNA
U6 rRNA
7SL rRNA
Actin
HepG2
G
Ctrl
EGR1 shRNA
EGR shRNA & PTEN shRNA
Total cell numbers（*10^5）
15
10
5
0
0 1 2 3 4 5
Time(day)
*
HepG2
H
Ctrl shRNA
EGR1 shRNA
EGR1-PTEN shRNA
OD450value
3.0
2.0
1.0
0.0
1 2 3 4
Time(day)
*
HepG2

Figure S6. PTEN silence reversed the inhibition of Pol III-directed transcription and cell proliferation caused by EGR1 down-regulation. (A) Western blot results showing the generation of HepG2 cell lines stably expressing both EGR1 shRNA and EGFP-RhoA. (B) RhoA-EGFP expression reverse the inhibition of Pol III product expression caused by EGR1 silence in HePG2 cells. RT-qPCR was performed using the HepG2 cell lines generated in A. (C) and (D) RhoA-EGFP expression did not affected the inhibition of cell proliferation cause by EGR down-regulation. Cell counting and CCK-8 assays were performed using the cell lines generated in A. (E) Western blot data showing the generation of HepG2 cell lines stably expressing both EGR1 shRNA and PTEN shRNA. (F) PTEN silence reversed the inhibition of Pol III product expression caused by EGR1 depletion in HepG2 cells. (G) and (H) PTEN depletion alleviated the inhibition of HepG2 cell proliferation caused by EGR1 depletion. Cell proliferation assays were performed using cell counting (G) and CCK-8 (H) methods, where the HepG2 cell lines expressing both EGR1 shRNA and PTEN shRNA or EGR1 shRNA only and the control cell line were examined. Each column or point in B-D and F-H represents the mean $\pm$ SD of three biological replicates (n=3). *, $p < 0.05$; **, $p < 0.01$. *P* values for C, D, G and H were obtained by two-way ANOVA; *p* values for B and F were obtained by one-way ANOVA, followed by Bonferroni test through comparing two groups within multiple groups. E/G, P/G and R/G in A and E represent the ratio of EGR1 (E), PTEN(P) and RhoA (R) intensities to the GAPDH (G) intensity, respectively (n=3).

Fig. S7

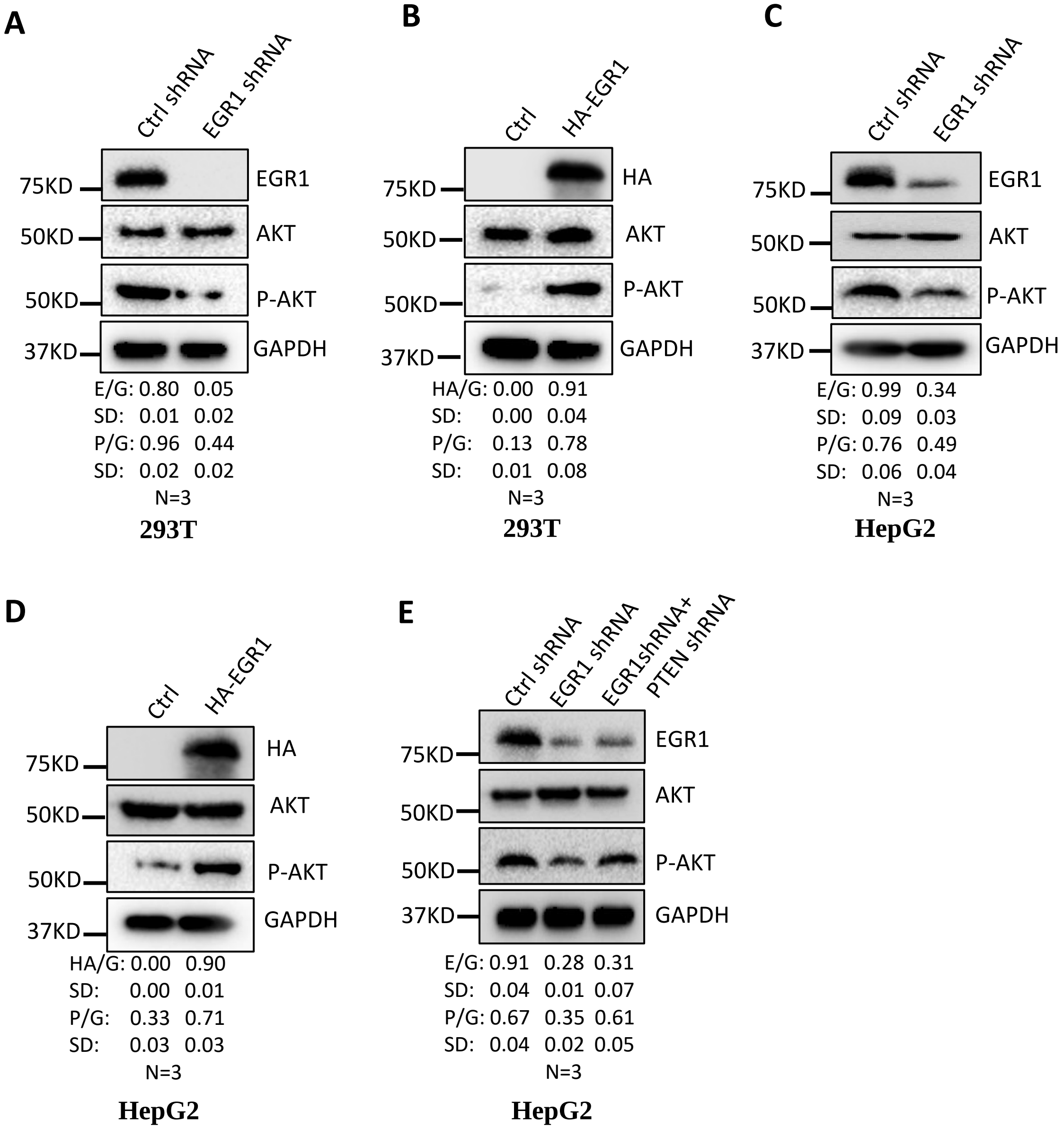

Figure S7. EGR1 regulates PTEN/AKT signaling pathway. (A) EGR1 depletion reduced AKT phosphorylation but not affect AKT expression. Western blot was performed using 293T cell lines expressing EGR1 shRNA or control shRNA and the antibodies against the factors as indicated. (B) EGR1 overexpression enhanced AKT phosphorylation. Western blot was performed using a 293T cell line expressing HA-EGR1 and its control cell line and the antibodies against the factors as indicated. (C) Western blot data showing the effect of EGR1 knockdown on AKT phosphorylation in HepG2 cells. (D) Western blot showing the effect of EGR1 overexpression on AKT phosphorylation in HepG2 cells. (E) Western blot showing the effect of both EGR1 and PTEN down-regulation on AKT phosphorylation in HepG2 cells. Western blot performed using the cell lines and the antibodies against the factors as indicated. E/G, H/G and P/G respectively represent the ratio of EGR1 (E), HA-EGR1(H), P-AKT(P) intensities to the GAPDH (G) intensity (n=3).

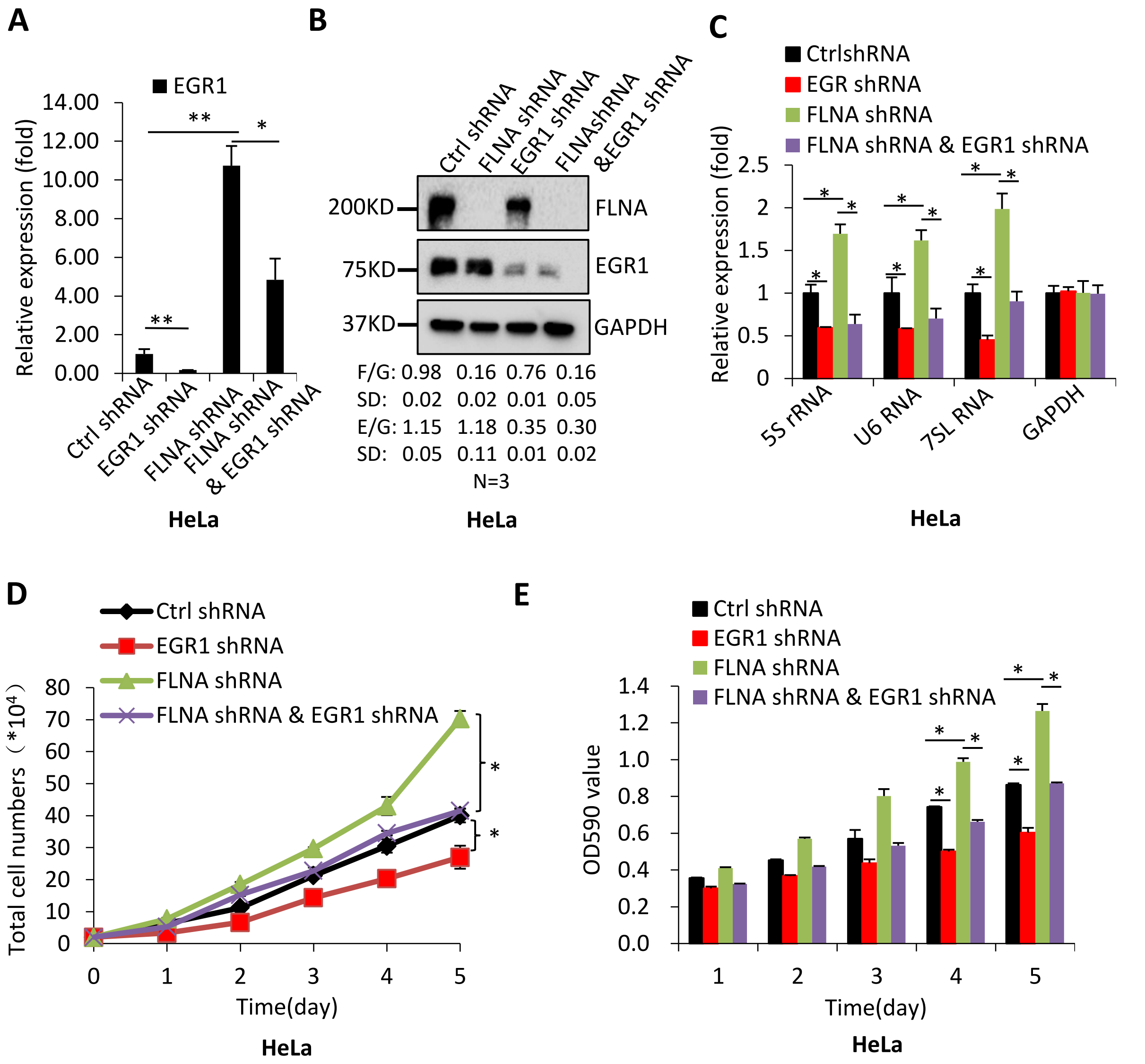

Figure S8. EGR1 down-regulation reduced activation of Pol III-directed transcription and cell proliferation caused by FLNA depletion. (A) A RT-qPCR result showing the generation of a HeLa cell line expressing both FLNA shRNA and EGR1 shRNA. (B) Western blot verified the HeLa cell line expressing both FLNA shRNA and EGR1 shRNA and its control cell line. F/G and E/G respectively represent the ratio of FLNA(F) and EGR1 (E) intensities to the GAPDH (G) intensity (n=3). (C) RT-qPCR results showing the effect of EGR1 silence on Pol III- directed transcription in the cell line with FLNA silence. (D) and (E) EGR1 silence inhibited the enhancement of HeLa cell lines caused by FLNA down-regulation. HeLa cell proliferation assays were performed by cell counting (D) and MTT (E) assays. Each column or point in A and C-E represents the mean±SD of three biological replicates (n=3). *, $p < 0.05$; **, $p < 0.01$. *P* values for A were obtained by a student’s *t*-test performed with two groups as indicated; *p* values for D and E were obtained by two-way ANOVA; *p* values for C were obtained by one-way ANOVA, followed by Bonferroni test through comparing two groups within multiple groups.

Table S1. The primer terms and sequences were used for gene expression analysis by RT-qPCR

Primer terms	Directions	Sequences
RT-tRNA-met	Forward	AACAACAACAACAACAACAA
	Reverse	TTAGCAGAGGATGGTTTA
RT-tRNA-Cys	Forward	GGGGGTATAGCTCAGGTG
	Reverse	AGGGGGCACCCGGATTTGA
RT-tRNA-Asp	Forward	TCCTCGTTAGTATAGTGG
	Reverse	CTCCCCGTCGGGGAATCG
RT-tRNA-Glu	Forward	TCCCTGGTGGTCTAGTGGA
	Reverse	TTCCCTGACCGGGAATCGA
RT-tRNA-Gly	Forward	GCGTTGGTGGTATAGTGG
	Reverse	TGCGTTGGCCGGGAATCG
RT-tRNA-His	Forward	GCCGTGATCGTATAGTGG
	Reverse	TGCCGTGACTCGGATTCG
RT-tRNA-Lys	Forward	GCCCGGATAGCTCAGTCG
	Reverse	CGCCCGAACAGGGACTTG
RT-tRNA-Leu	Forward	GGTAGCGTGGCCGAGC
	Reverse	TGGCAGCGGTGGGATTC
RT-tRNA-Gln	Forward	GGTTCCATGGTGTAATGG
	Reverse	AGGTTCCACCGAGATTTG
RT-tRNA-Thr	Forward	GGCTCCATAGCTCAGGG
	Reverse	AGGCCCCAGCGAGATTTG
RT-tRNA-Val	Forward	TGTTTCCGCCCGGTTTCG
	Reverse	GTTTCCGTAGTGTAGTGG
RT-tRNA-Trp	Forward	GACCTCGTGGCGCAACGGT
	Reverse	TGACCCCGACGTGATTCGA
RT-tRNA-Tyr	Forward	CCTTCGATAGCTCAGCTGGTAG
	Reverse	CGGAATTGAACCAGCCGACCTAA
RT-5S rRNA	Forward	CTACGGCCATACCACCCT
	Reverse	GCCTACAGCACCCGGTATT
RT-U6 RNA	Forward	CTCGCTTCGGCAGCACATA
	Reverse	ATATGGAACGCTTATCACG
RT-7SL RNA	Forward	ACTAAGTTCGGCATCAATA
	Reverse	GAGTGCAGTGGCTATTCA
